# Associations of air pollutant concentrations with longitudinal kidney function changes in patients with chronic kidney disease

**DOI:** 10.1038/s41598-023-36682-4

**Published:** 2023-06-13

**Authors:** Cheng-Yin Chung, Shang-Yu Wu, Huei-Hsuan Chiu, Tzu-Ning Wu, Your-Tong Wang, Ming-Yen Lin, on behalf of the iH3 Research Group

**Affiliations:** 1 Department of Internal Medicine, Pingtung Hospital, Ministry of Health and Welfare, Pingtung, 900214 Taiwan; 2 Division of Nephrology, Department of Internal Medicine, Kaohsiung Medical University Hospital, Kaohsiung Medical University, No. 100, TzYou 1st Road, Sanmin District, Kaohsiung City, 80708 Taiwan; 3 Department of Kidney Care, College of Medicine, Kaohsiung Medical University Hospital, Kaohsiung Medical University, Kaohsiung, 80708 Taiwan; 4 Department of Nursing, Ministry of Health and Welfare, Pingtung Hospital, Pingtung, 900214 Taiwan

**Keywords:** Kidney diseases, Public health, Environmental sciences, Nephrology

## Abstract

This longitudinal cohort study investigated the associations of air pollutant exposures, including CO, NO, NO_2_, NO_x_, O_3_, PM_10_, PM_2.5_, and SO_2_, with long-term kidney function changes in patients with chronic kidney disease (CKD). We enrolled 447 CKD patients who took part in a universal hospital pre-ESRD care program during 2011–2015. The daily average air pollutant exposures and temperature were estimated for each patient, with different levels of air pollutant concentrations defined by 5-knot and restricted cubic spline function. Predicted annual estimated glomerular filtration (eGFR) slope values by one mixed model were considered as the study outcome. The average age of the study population was 77.1 ± 12.6 years, and the median annual eGFR decreased by 2.1 ml/min/1.73 m^2^ per year from 30 ml/min/1.73 m^2^ at baseline during a mean follow-up time of 3.4 years. The univariable and multivariable analyses revealed no significant linear and non-linear associations between 5-knot air pollutant concentrations and annual eGFR slope. In addition, the visualized spline effect plots show insignificant variation patterns in annual eGFR slope values with increased air pollutant concentrations. These results encourage more extensive studies to clarify the causal relationships and mechanisms of long-term specific air pollutant exposures and longitudinal kidney function change, especially in CKD populations.

## Introduction

Ambient air pollution threats to human health have become more severe over the past few decades due to economic development and urbanization. In 2013, it was estimated that over 85% of the world’s population lived in areas where fine particles (particulate matter < 2.5 μm in aerodynamic diameter [PM_2.5_]) exceeded the WHO 2005 regulatory standard of 10 g/m^3^^1,3^. Poor ambient air conditions have significantly impacted human life and productivity loss. Based on the Global Burden of Disease 2015 report, annual mortality attributed to ambient air pollution increased to 4.2 million deaths from fewer than 1 million in 2000^2^. Even in the U.S., a developed country, there were approximately 47,000 deaths related to exposure to human cause ambient PM_2.5_ in 2019, 37% of which was directly related to fossil fuel burning^3^. Air pollution could cause more people to die due to inappropriate literacy, low awareness, and insufficient protection in a developing country. In 2017, air pollution in China resulted in nearly 1.24 million deaths, the equivalent of 1513 per 100,000 disability-adjusted life-year rates^4^. Therefore, understanding the health influences of air pollution is vital to develop appropriate preventive actions.

Previous scientific evidence has highlighted that ambient air pollution causes several direct and indirect adverse health effects. It is well known that poor air conditions trigger acute respiratory diseases such as asthma and allergic rhinitis, and long-term lung damage may elevate chronic obstructive pulmonary disease and lung cancer risks^5^. Moreover, air pollutants may increase obesity^6^, diabetes^7^, and cardiovascular disease risks^8^ by disturbing the human immune and endocrine systems. In addition, long-term exposure to air pollutants is linked to kidney disease development. A recent systematic review of thirteen studies of the effects of air pollutants on kidney disease over the past two decades^9^ supported that air particle matter, nitrogen dioxide (NO_2_), and carbon monoxide (CO) are associated with CKD development, but the results should be interpreted with caution due to the considerable study heterogeneity^9^.

Taiwan has a high density of road networks and industries that could lead to poor ambient air quality. During the fall and winter seasons, Environmental Protection Administration Executive Yuan usually warns of poor ambient air conditions for vulnerable populations in southern areas^10^. Previous studies have proposed several factors, such as insurance systems, herbs containing aristolochic acid, and specific infections that are associated with the highest incidence of end-stage kidney disease (ESKD) globally^11–15^. Evidence of environmental pollutants' roles in long-term kidney disease progression remains scarce. Some studies in Taiwan have reported that specific air pollutants are associated with an increased risk of reduced kidney function^16,17^, CKD, and ESKD^18^ in the general population, as well as kidney function decline^19,20^ and progression to kidney failure^21^ in CKD patients. However, it is unclear whether long-term kidney function change in patients with CKD is affected by air conditions when they live in high air pollution areas. This study explored different air pollutant effects on long-term kidney function deterioration in southern Taiwan.

## Results

### Patient and ambient air pollutant characteristics

**Figure 1:**
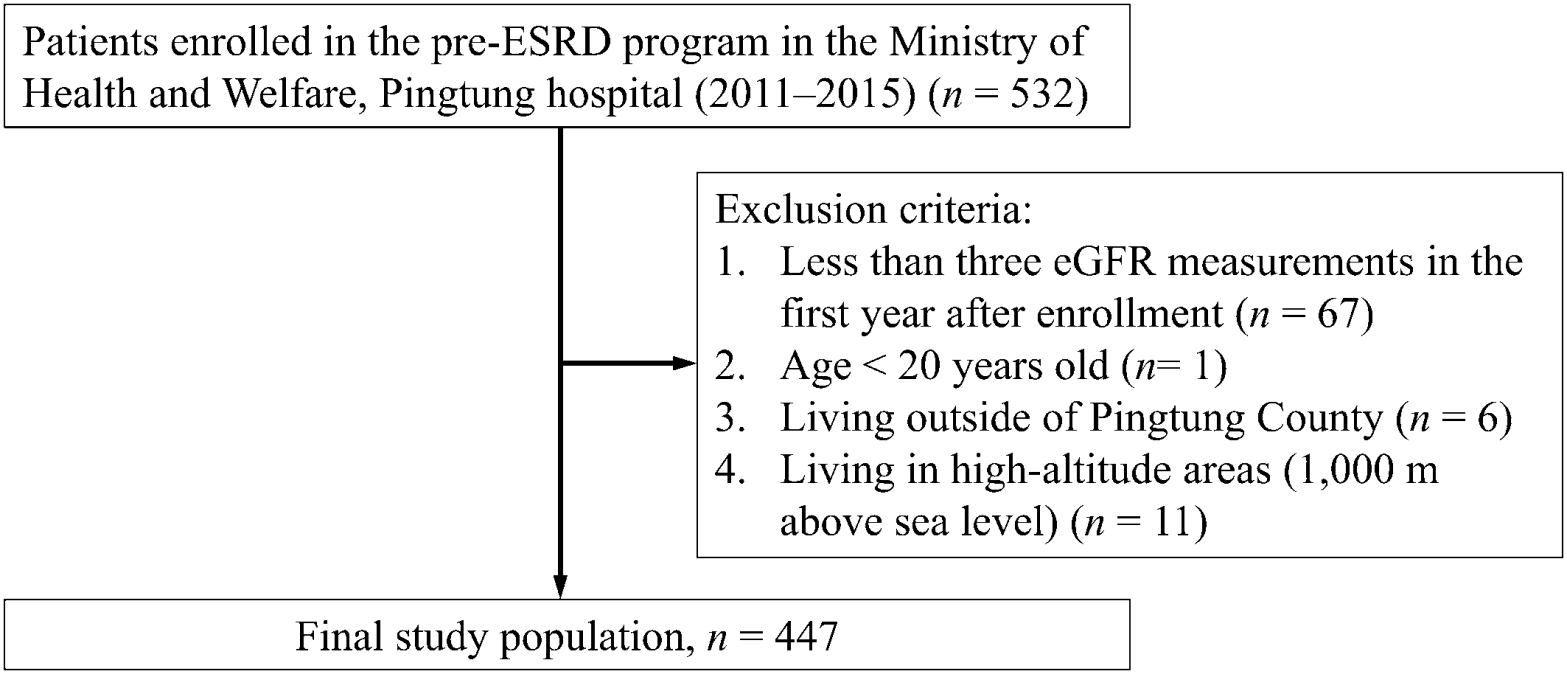
Study flow chart. Abbreviations: ESRD, end-stage renal disease; eGFR, estimated glomerular filtration rate. Patients living in the high-altitude villages, namely Sandimen, Wutai, Majia, Laiyi, and Taiwu, were excluded from the study.

After excluding patients who had fewer than three estimated glomerular filtration rate (eGFR) measurements in their first year after enrollment (n = 75), as well as those under the age of 20 (n = 1), those who lived outside of Pingtung city (n = 6), and those who lived in high-altitude areas (n = 11), a total of 447 patients with CKD were enrolled in this study (Fig. 1). The patients’ mean age was 77.2; three-fourths of them had hypertension, and nearly half of them had diabetes mellitus. Over half of the eGFR measurements at enrollment were below 30.0 ml/min/1.73 m^2^, and most (over 80%) were accompanied by proteinuria (Table 1). The air pollutant average concentrations of coarse particulate matter (particulate matter < 10 μm in aerodynamic diameter [PM_10_] (64.31 ± 1.13) and PM_2.5_ (35.75 ± 0.90) in the participants were much higher than the 2021 annual air quality standard set by the WHO^22^, which was 15 μg/m^3^ and 5 μg/m^3^, respectively. The patient characteristics by quartile of air pollutants are provided in Supplementary Tables S1–S8). In general, those who were old with a high proportion of high educational years, low prevalence of hypertension, and a low proportion of high rank of urine protein creatinine ratio (UPCR) were significantly more likely to be exposed to the highest quartile of most air pollutants (CO, NO_2_, nitrogen oxide [NO_x_], PM_10_, and PM_2.5_) concentrations. However, the highest quartile group of sulfur dioxide (SO_2_) concentration had a significantly lower proportion of the high educational years, more prevalent angiotensin-converting enzyme inhibitors or angiotensin II receptor blockers (ACEI/ARB) use, and a higher proportion of high-rank UPCR. Only a significant difference in hypertension prevalence was found between quartiles of nitrogen monoxide (NO) concentration, and only an educational level difference was found between quartiles of ozone (O_3_) concentration.

**Table 1:** Patient characteristics.

Characteristics	Overall (n = 447)
Age, yrs	77.1 ± 12.6
Gender	
Men	269 (60.2)
Women	178 (40.0)
Smoking	31 (7.1)
Alcohol consumption	26 (5.9)
Drugs (missing n = 36)	
NSAIDs	12 (3.0)
ACEI/ARB	211 (52.2)
Co-morbidity	
Diabetes mellitus	206 (46.8)
Hypertension	331 (75.2)
Cerebrovascular accident	39 (8.9)
Congestive heart failure	36 (8.2)
Ischemic heart disease	32 (7.3)
Gout	45 (10.2)
Cancer	16 (3.6)
eGFR at enrollment, ml/min/1.73m^2^	29.7 (19.6; 37.3)
≥ 45	33 (7.38)
30–45	183 (40.94)
15–29	165 (36.91)
< 15	66 (14.77)
Urine protein creatinine ratio, mg/g	759 (230; 1,754)
< 150	74 (16.55)
150–1000	182 (40.72)
1000–2999	133 (29.75)
> 3000	58 (12.98)
Annual eGFR slope, ml/min/1.73 m^2a^	− 2.1 (− 3.4; − 0.9)
Exposure estimation from air quality monitoring stations	
CO, ppm	0.46 ± 0.03
NO, ppb	2.42 ± 0.07
NO_2_, ppb	13.38 ± 0.72
NO_x_, ppb	15.77 ± 0.73
O_3_, ppb	31.48 ± 0.29
PM_10_, μg/m^3^	64.31 ± 1.13
PM_2.5_, μg/m^3^	35.75 ± 0.90
SO_2_, ppb	3.63 ± 0.22
Ambient temperature, °C	25.33 ± 0.10

ppb, parts per million; ppm, parts per billion; μg/m^3^, micrograms per cubic meter; NSAIDs, non-steroidal anti-inflammatory drugs; ACEI, angiotensin-converting enzyme inhibitor; ARB, angiotensin II receptor blocker; eGFR, estimated glomerular filtration rate; CO, carbon monoxide; NO, nitrogen monoxide; NO_2_, nitrogen dioxide; NOx, nitrogen oxide; O_3_, ozone; PM_10_, ambient coarse particulate matter ($\le$ 10 μm); PM_2.5_, ambient coarse particulate matter ($\le$ 2.5 μm); SO_2_, sulfur dioxide.

^a^The subjects’ annual eGFR slope was estimated by one mixed model with random intercept and slope.

### Associations of air pollutant concentration with annual eGFR slope

**Figure 2:**
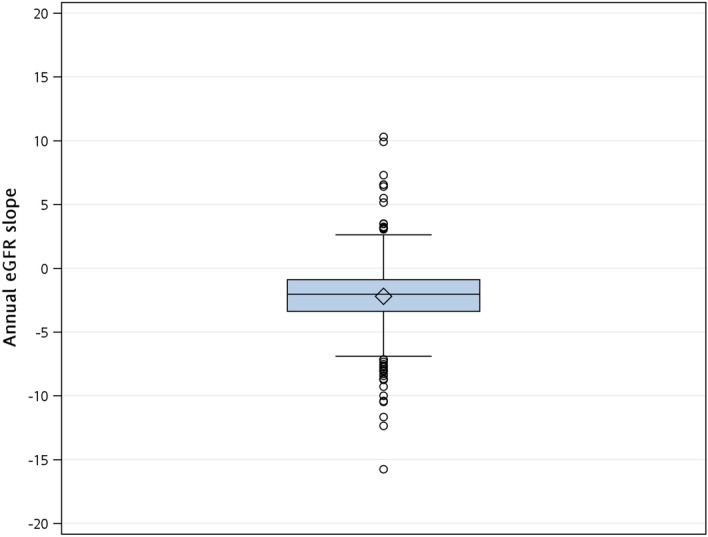
The Box plot of the estimated annual eGFR slope. The estimated annual eGFR slope was obtained by a mixed model with random intercept and slope.

**Figure 3:**
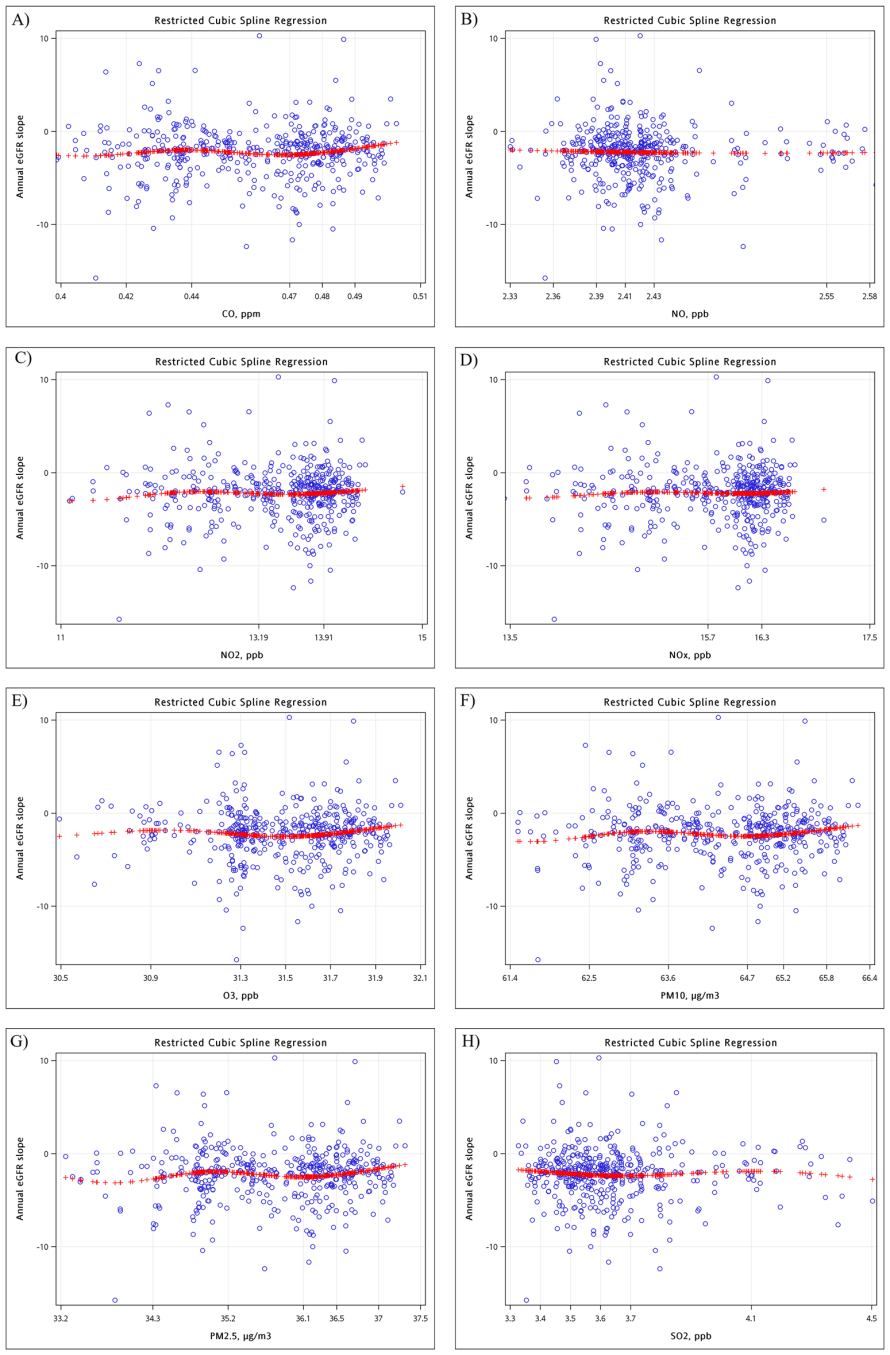
The associations of (**A**) CO, (**B**) NO, (**C**) NO_2_, (**D**) NO_X_, (**E**) O_3_, (**F**) PM_10_, (**G**) PM_2.5_, and (**H**) SO_2_ values with fitted annual eGFR slope. Abbreviations: ppb, parts per million; ppm, parts per billion; μg/m^3^, micrograms per cubic meter; CO, carbon monoxide; NO, nitrogen monoxide; NO_2_, nitrogen dioxide; NOx, nitrogen oxide; O_3_, ozone; PM_10_, particulate matter < 10 μm in aerodynamic diameter; PM_25_, particulate matter < 2.5 μm in aerodynamic diameter; SO_2_, sulfur dioxide; eGFR, estimated glomerular filtration rate. Predicted values were generated by univariable general linear models with restricted cubic splines.

During the observation period, 5,811 eGFR measurements were recorded showing that the median annual eGFR decreased by 2.1 ml/min/1.73 m^2^ per year with the interquartile range: 0.9, 3.4 reduction per year (Table 1, Fig. 2, and Supplementary Fig. S1). The cubic spline plots imply non-linear relationships between long-term eGFR change and the quartile of air pollutant concentration (Supplementary Fig. S2). In the modelings, we first determined 5-knot of air pollutants for all models because they produced smaller Akaike information criterion (AIC) values. There were no significant differences in the 5-knot groups of any air pollutant concentrations associated with annual eGFR slope after putting a group of air pollutant concentrations in the model (Table 2). Even adjusting for age, sex, educational level, diabetes mellitus, hypertension, cerebrovascular accident, congestive heart failure, ischemic heart disease, gout, ACEI/ARB, temperature, and log (UPCR), no selected air pollutant concentrations significantly associated with annual eGFR slope (The ranges of Type III sum of squares *P*-values: 0.05–0.26). After transforming the air pollutant concentrations by a 5-knot restricted cubic spline function, model one’s results in Table 3 neither support significant non-linear relationships between air pollutants concentrations and long-term kidney function changes. Similar findings were demonstrated in the model, further adjusted by the selected covariates. The visualized spline effect plots show insignificant variation patterns in predicted annual eGFR slope values with increased air pollutants' concentrations (Fig. 3).

**Table 2:** Association of five-knot group of air pollutant concentration with annual eGFR slope by a linear regression model.

	Model 1		*P*-value	Model 2		*P*-value
	Regression coefficient	95% CI		Regression coefficient	95% CI	
CO, ppm			0.33			0.08
< 0.42	0.00 [Reference]	–		0.00 [Reference]	–	
0.42 ≤ CO < 0.44	0.28	(− 0.91–1.46)	0.65	0.57	(− 0.88–2.01)	0.44
0.44 ≤ CO < 0.47	0.16	(− 1.05–1.37)	0.80	0.47	(− 1.71–2.66)	0.67
0.47 ≤ CO < 0.48	− 0.16	(− 1.35–1.03)	0.79	1.32	(− 1.58–4.22)	0.37
0.48 ≤ CO < 0.49	0.78	(− 0.44–2.00)	0.21	2.38	(− 1.00–5.76)	0.17
≥ 0.49	0.36	(− 1.28–1.99)	0.67	2.08	(− 1.93–6.08)	0.31
NO, ppb			0.23			0.14
< 2.36	0.00 [Reference]	–		0.00 [Reference]	–	
2.36 ≤ NO < 2.39	0.67	(− 0.53–1.86)	0.27	0.62	(− 0.81–2.04)	0.40
2.39 ≤ NO < 2.41	0.93	(− 0.25–2.12)	0.12	0.87	(− 0.51–2.25)	0.21
2.41 ≤ NO < 2.43	0.27	(− 0.90–1.44)	0.65	0.07	(− 1.22–1.36)	0.92
2.43 ≤ NO < 2.55	0.01	(− 1.18–1.20)	0.98	− 0.18	(− 1.44–1.08)	0.78
≥ 2.55	0.62	(− 0.92–2.16)	0.43	− 0.02	(− 1.52–1.47)	0.98
NO_2_, ppb			0.69			0.15
< 12.15	0.00 [Reference]	–		0.00 [Reference]	–	
12.15 ≤ NO_2_ < 13.19	0.18	(− 0.98–1.34)	0.76	0.05	(− 1.18–1.28)	0.93
13.19 ≤ NO_2_ < 13.69	− 0.07	(− 1.25–1.11)	0.91	− 0.87	(− 2.33–0.59)	0.24
13.69 ≤ NO_2_ < 13.91	− 0.20	(− 1.37–0.96)	0.73	− 0.21	(− 1.97–1.55)	0.82
13.91 ≤ NO_2_ < 14.21	0.44	(− 0.74–1.63)	0.46	0.14	(− 1.84–2.13)	0.89
≥ 14.21	− 0.14	(− 1.80–1.53)	0.87	− 0.35	(− 2.71–2.02)	0.77
NO_x_, ppb			0.45			0.3
< 14.53	0.00 [Reference]	–		0.00 [Reference]	–	
14.53 ≤ NO_X_ < 15.66	0.25	(− 0.89–1.39)	0.66	− 0.05	(− 1.25–1.16)	0.94
15.66 ≤ NO_X_ < 16.10	0.13	(− 1.04–1.29)	0.83	− 0.79	(− 2.22–0.64)	0.28
16.10 ≤ NO_X_ < 16.28	− 0.33	(− 1.48–0.82)	0.57	− 0.69	(− 2.35–0.96)	0.41
16.28 ≤ NO_X_ < 16.54	0.51	(− 0.65–1.67)	0.39	− 0.13	(− 2.01–1.75)	0.90
≥ 16.54	− 0.01	(− 1.69–1.68)	0.99	− 0.53	(− 2.67–1.62)	0.63
O_3_, ppb			0.47			0.25
< 30.92	0.00 [Reference]	–		0.00 [Reference]	–	
30.92 ≤ O_3_ < 31.31	− 0.20	(− 1.48–1.07)	0.75	− 0.16	(− 1.35–1.03)	0.79
31.31 ≤ O_3_ < 31.53	− 0.13	(− 1.41–1.15)	0.85	0.33	(− 0.98–1.65)	0.62
31.53 ≤ O_3_ < 31.71	− 0.53	(− 1.81–0.74)	0.41	0.76	(− 0.93–2.45)	0.38
31.71 ≤ O_3_ < 31.89	0.31	(− 0.99–1.61)	0.64	1.77	(− 0.19–3.72)	0.08
≥ 31.89	0.06	(− 1.64–1.75)	0.95	1.58	(− 0.89–4.04)	0.21
PM_10_, μg/m^3^			0.38			0.05
< 62.45	0.00 [Reference]	–		0.00 [Reference]	–	
62.45 ≤ PM_10_ < 63.62	0.54	(− 0.69–1.76)	0.39	0.86	(− 0.33–2.05)	0.15
63.62 ≤ PM_10_ < 64.71	0.54	(− 0.71–1.78)	0.40	0.80	(− 0.57–2.17)	0.25
64.71 ≤ PM_10_ < 65.22	0.20	(− 1.03–1.43)	0.75	1.64	(− 0.07–3.35)	0.06
65.22 ≤ PM_10_ < 65.84	1.04	(− 0.22–2.29)	0.10	2.60	(0.62–4.58)	0.01
≥ 65.84	0.65	(− 1.01–2.32)	0.44	2.32	(− 0.18–4.83)	0.07
PM_2.5_, μg/m^3^			0.36			0.07
< 34.31	0.00 [Reference]	–		0.00 [Reference]	–	
34.31 ≤ PM_25_ < 35.15	0.72	(− 0.57–2.01)	0.27	0.89	(− 0.36–2.15)	0.16
35.15 ≤ PM_25_ < 36.06	0.69	(− 0.62–2.00)	0.30	1.11	(− 0.36–2.57)	0.14
36.06 ≤ PM_25_ < 36.49	0.35	(− 0.95–1.64)	0.60	1.89	(0.04–3.74)	0.05
36.49 ≤ PM_25_ < 36.99	1.17	(− 0.15–2.49)	0.08	2.85	(0.73–4.98)	0.01
≥ 36.99	0.80	(− 0.91–2.52)	0.36	2.65	(− 0.01–5.32)	0.05
SO_2_, ppb			0.65			0.26
< 3.38	0.00 [Reference]	–		0.00 [Reference]	–	
3.38 ≤ SO_2_ < 3.50	0.80	(− 0.60–2.20)	0.26	1.07	(− 0.36–2.5)	0.14
3.50 ≤ SO_2_ < 3.58	0.27	(− 1.11–1.65)	0.70	0.63	(− 0.75–2.00)	0.37
3.58 ≤ SO_2_ < 3.67	0.17	(− 1.22–1.55)	0.81	0.02	(− 1.34–1.39)	0.97
3.67 ≤ SO_2_ < 4.09	0.24	(− 1.15–1.63)	0.74	0.01	(− 1.41–1.43)	0.99
≥ 4.09	0.50	(− 1.19–2.19)	0.56	< 0.01	(− 1.68–1.69)	1.00

CO, carbon monoxide; NO, nitrogen monoxide; NO_2_, nitrogen dioxide; NO_x_, nitrogen oxide; O_3_, ozone; PM_10_, particulate matter < 10 μm in aerodynamic diameter; PM_25_, particulate matter < 2.5 μm in aerodynamic diameter; SO_2_, sulfur dioxide; ppb, parts per billion; ppm, parts per million; μg/m^3^, micrograms per cubic meter; Q, quartile; CI, confidence interval.

All studied air pollutant concentrations were categorized by their percentiles: 5, 27.5, 50, 72.5, and 95. Model 1 is estimated by linear regression model after putting a group of air pollutant concentrations. Independent variables in Model 2 are listed as independent variables in model 1, and age, sex, educational level, diabetes mellitus, hypertension, cerebrovascular accident, congestive heart failure, ischemic heart disease, gout, angiotensin-converting enzyme inhibitor/ angiotensin II receptor blocker, temperature, and log (urine protein creatinine ratio). The Type III tests of fixed effect P-value was shown at the same raw adjacency to the listed name of the air pollutant.

**Table 3:** Association of air pollutant concentration with annual eGFR slope by a linear regression model with restricted cubic splines.

	Model 1		*P*-value	Model 2		*P*-value
	Regression coefficient	95% CI		Regression coefficient	95% CI	
CO, ppm						
β_0_	15	(− 38–68)	0.57	187	(− 206–580)	0.35
Spl_β_1_	− 45	(− 177–87)	0.50	− 41	(− 165–84)	0.52
Spl_β_2_	2358	(− 2233–6948)	0.31	2037	(− 2697–6771)	0.40
Spl_β_3_	− 5475	(− 14,429–3480)	0.23	− 4307	(− 13,390–4776)	0.35
Spl_β_4_	4988	(− 1216–11,191)	0.11	3781	(− 2394–9956)	0.23
NO, ppb						
β_0_	6	(− 28–41)	0.72	54	(− 16–125)	0.13
Spl_β_1_	− 4	(− 18–11)	0.63	− 6	(− 22–9)	0.42
Spl_β_2_	10	(− 117–138)	0.88	2	(− 134–138)	0.98
Spl_β_3_	4	(− 329–337)	0.98	49	(− 289–387)	0.77
Spl_β_4_	− 47	(− 444–349)	0.82	− 92	(− 470–287)	0.63
NO_2_, ppb						
β_0_	14	(− 29–56)	0.52	62	(− 97–220)	0.44
Spl_β_1_	− 2	(− 5–2)	0.44	− 2	(− 5–2)	0.36
Spl_β_2_	2	(− 2–6)	0.31	2	(− 2–6)	0.25
Spl_β_3_	− 5	(− 13–4)	0.28	− 5	(− 13–3)	0.19
Spl_β_4_	3	(− 3–9)	0.26	5	(− 1–10)	0.12
NO_x_, ppb						
β_0_	11	(− 33–55)	0.63	15	(− 118–148)	0.82
Spl_β_1_	− 1	(− 4–2)	0.54	− 1	(− 4–2)	0.52
Spl_β_2_	1	(− 2–4)	0.43	1	(− 2–4)	0.45
Spl_β_3_	− 3	(− 8–3)	0.40	− 3	(− 8–3)	0.36
Spl_β_4_	2	(− 3–7)	0.39	3	(− 2–7)	0.22
O_3_, ppb						
β_0_	− 57	(− 421–307)	0.76	222	(− 135–579)	0.22
Spl_β_1_	2	(− 10–14)	0.77	− 1	(− 12–11)	0.92
Spl_β_2_	0	(− 29–29)	0.99	1	(− 28–30)	0.95
Spl_β_3_	− 6	(− 63–50)	0.82	− 1	(− 60–59)	0.99
Spl_β_4_	14	(− 25–52)	0.48	2	(− 40–45)	0.91
PM_10_, μg/m^3^						
β_0_	82	(− 95–260)	0.36	155	(− 86–396)	0.21
Spl_β_1_	− 1	(− 4–2)	0.34	− 1	(− 4–2)	0.45
Spl_β_2_	2	(− 1–4)	0.17	1	(− 1–3)	0.26
Spl_β_3_	− 3	(− 8–1)	0.12	− 3	(− 7–2)	0.21
Spl_β_4_	3	(0–6)	0.06	2	(− 1–6)	0.12
PM_2.5_, μg/m^3^						
β_0_	73	(− 39–186)	0.20	162	(− 74–397)	0.18
Spl_β_1_	− 2	(− 6–1)	0.18	− 2	(− 5–2)	0.36
Spl_β_2_	3	(0–7)	0.08	2	(− 1–6)	0.20
Spl_β_3_	− 7	(− 14–0)	0.05	− 5	(− 12–2)	0.17
Spl_β_4_	5	(1–10)	0.03	4	(− 1–9)	0.11
SO_2_, ppb						
β_0_	− 7	(− 33–18)	0.57	81	(− 7–170)	0.07
Spl_β_1_	2	(− 6–10)	0.63	1	(− 7–10)	0.80
Spl_β_2_	− 9	(− 28–9)	0.33	− 5	(− 24–15)	0.62
Spl_β_3_	22	(− 16–61)	0.25	9	(− 30–48)	0.65
Spl_β_4_	− 21	(− 54–11)	0.20	− 4	(− 36–29)	0.83

CO, carbon monoxide; NO, nitrogen monoxide; NO_2_, nitrogen dioxide; NO_x_, nitrogen oxide; O_3_, ozone; PM_10_, particulate matter < 10 μm in aerodynamic diameter; PM_25_, particulate matter < 2.5 μm in aerodynamic diameter; SO_2_, sulfur dioxide; ppb, parts per billion; ppm, parts per million; μg/m^3^, micrograms per cubic meter; Q, quartile; CI, confidence interval.

The 5-knot (percentile: 5, 27.5, 50, 72.5, and 95) restricted cubic spline function was determined for all studied air pollutant concentrations. The knots for CO, NO, NO_2_, NO_X_, O_3_, PM_10_, PM_2.5_, and SO_2_ are (0.42, 0.44, 0.47, 0.48, and 0.49), (2.36, 2.39, 2.41, 2.43, and 2.55), (12.15, 13.19, 13.69, 13.91, and 14.21), (14.53, 15.66, 16.10, 16.28, and 16.54), (30.92, 31.31, 31.53, 31.71, and 31.89), (62.45, 63.62, 64.71, 65.22, and 65.84), (34.31, 35.15, 36.06, 36.49, and 36.99), and (3.38, 3.5, 3.58, 3.67, and 4.09), respectively. β_0_ is a coefficient of intercept, and Spl_β_1_- Spl_β_4_ are coefficients for the restricted cubic spline functions of studied air pollutant concentrations. Model 1 is estimated by a general linear model for the annual estimated glomerular filtration rate slope after only putting one restricted cubic spline function of studied air pollutant concentration. Independent variables in Model 2 are listed as the independent variable in model 1, and age, sex, educational level, diabetes mellitus, hypertension, cerebrovascular accident, congestive heart failure, ischemic heart disease, gout, angiotensin-converting enzyme inhibitor/ angiotensin II receptor blocker, temperature, and log (urine protein creatinine ratio).

## Discussion

In this retrospective cohort study, we traced 447 CKD patients who regularly received care in one hospital. After a mean follow-up of 3.4 years, each patient’s annual eGFR slope was estimated from 5811 consequent eGFR measurements. We surprisedly found that 5-knot linear and 5-knot restricted cubic splines models findings did not support that exposure to different CO, NO, NO_2_, NO_X_, O_3_, PM_10_, PM_2.5_, and SO_2_ concentrations were significantly associated with varying annual eGFR slope among patients with advanced CKD.

There is a shortage of evidence regarding the types of ambient air pollutants and which doses of those pollutants can lead to kidney function change, especially in the CKD population. Most previous studies have mainly focused on deterministic kidney outcomes. Lin et al. applied claim data to explore the effects of air pollution exposure on the incidence of CKD or ESKD in the general population, showing that high concentrations of air pollutants (NO, NO_2_, NOx, PM_2.5,_ and SO_2_) increased CKD and ESKD intensities^18^. Another study used a clinical database with a more comprehensive confounder collection and demonstrated that PM_2.5_ concentration significantly increased (by nearly 20%) kidney replacement therapy incidence in patients with advanced CKD^21^. Moreover, a recent study defined two consequent eGFR measurements which declined by more than 25% from the baseline within one year as kidney function deterioration. Almost all air pollutants, except O_3_, were significantly associated with kidney function deterioration in the CKD cohort^20^. In addition, another study observed that PM_2.5_ and NO_2_ exposure with a mean 2.3 years follow-up was associated with an increased incidence of first hitting eGFR reduction by more than 5 ml/min/1.73 m^2^/year^19^. Compared with the above studies, the present study recorded more eGFR measurements with a much longer duration, thus making it more appropriate for evaluating the associations between air pollution and long-term kidney function change. However, our results did not indicate that any air pollutant concentration was significantly associated with long-term kidney function deterioration. Indeed, our findings do not argue against the impacts of air pollutants on kidney health, but instead emphasize the importance of precisely arranging care resources in response to the influences of air pollution on regular kidney function measurement between various areas.

Certain factors may explain the current findings, which were different from the observations forwarded by the previous cohort studies involving advanced CKD patients. First, oxidative stress induced by PM, NO, NO_2_, and O_3_, could lead to inflammatory reactions, DNA damage, and organ dysfunction. The degree of oxidative reactions is a critical factor affecting pathogenic mechanisms in CKD progression^23^. In addition, the different compositions of PM may affect the degree of oxidative stress. The concentration of transition metals and organic carbon compounds within PM may be different in various areas, which can induce different degrees of oxidative reactions^24^, thus leading to various observations on CKD progression. Second, and similar to results from animal studies^25,26^, most previous studies observed the association using short-term rather than long-term kidney function declines. Indeed, employing this approach may bring into the picture rapid kidney function decline caused by other acute conditions, such as trauma and the use of nephrotoxin drugs, which occur more often in urban than rural areas; this would result in a higher likelihood of the above-mentioned association being falsely confirmed. Thirdly, it is not impossible that our study population was too homogeneous to observe the impacts of high air pollutant concentration on kidney function progression. The lowest air pollutant concentrations in the reference groups were higher than those in previous studies^19–21^, which may have weakened the associations. Finally, air pollutant-induced cardiovascular dysfunction in the study might be modified by regular care. Through this, it is then possible to offset the impact of air pollutants on kidney function decline. More research is needed to understand the mechanisms of air pollutants and their effect on long-term kidney function progression.

Our study observed potential negative associations between NO_x_ concentration and kidney function decline (Table 2). The mechanisms were unclear. Endogenous NO is a common intercellular messenger in all vertebrates and has physiological functions in blood flow regulation, inhibiting platelet adhesion and neuron activity^27^. In the kidney, NO modulates hemodynamics, medullary perfusion, pressure natriuresis, tubuloglomerular feedback, tubular sodium reabsorption, and the kidney sympathetic nerve^28^. Therefore, we speculate that ambient air pollutants, including PM, NO, and O_3_, as potent oxidants could generate superoxides that interact with endogenous NO to form peroxynitrite, thus resulting in lipid peroxidation, protein oxidation, inactivation of enzymes, worsening of NO insufficiency in cells, and impaired vascular relaxation, finally leading to cardiovascular dysfunction. Cardiorenal syndrome is a concept used to connect cardiovascular dysfunction to kidney function deterioration^29^. Our observation offers one possible explanation for this connection.

This study has several strengths. First, a much longer follow-up time allowed us to observe more long-term kidney function change than was possible in other studies. In addition, the patients were regularly cared for by the CKD care team, which ensured that our findings were not affected, or only affected to a limited degree by care quality. Third, we evaluated kidney function change almost per 3 months for each patient, which could have decreased the influences of acute kidney injury on eGFR slope estimation. Fourth, the laboratory data were examined in the same laboratory, thereby reducing experimental error. Finally, the high density of air quality stations within the study area increased the accuracy of individual air pollutant exposure estimations.

Nonetheless, there are limitations in this study. First, there may be some potential unmeasured confounding factors (either time-fixed or time-vary) associated with kidney function deterioration, such as detailed outdoor activities, income, and convenience of access to healthcare, which may have partially influenced our estimations. Second, the residential addresses may not have comprehensively reflected individual air pollutant exposure due to migration. Since work is the main reason for migration, we expected that these influences would be minor because most participants were elderly and retired. Third, the study averaged air pollutant values rather than directly using hourly raw data, so some information regarding short-term air pollutant effects on kidney function changes may be lost. Finally, our findings were based primarily on elderly patients with advanced CKD, so the results may not be generalizable to the whole CKD population.

In conclusion, this longitudinal study revealed that ambient air pollutant concentrations were not significantly associated with long-term kidney function decline in patients with advanced CKD. Although our findings did not support the modern mainstream contention, the findings encourage more extensive studies to clarify the causal relationships and mechanisms between long-term specific air pollutant exposures and longitudinal kidney function change, especially in vulnerable populations.

## Materials and methods

### Study design and population

This retrospective cohort study enrolled stage 3b to 5 CKD patients who were new participants in a universal national pre-ESRD care program at the Pingtung Hospital from 2011 to 2015. The above-mentioned regional hospital and another 16 hospitals offer acute and chronic medical services and hospital admission for nearly 50,400 residents in southern Taiwan. The participants all lived at a longitude between 120°42′ E and 120°85′ E and a latitude between 22°02′ N and 23°86′ N. The care program reimbursed nephrologists who organized care teams to deliver multidisciplinary care for patients with uncontrolled proteinuria or eGFR less than 45 ml/min/1.73 m^2,30,31^. The care teams regularly evaluated the patient's blood pressure and kidney functions, as well as proteinuria, and offered diet and kidney care knowledge, all of which allowed us to study long-term kidney function change. The Institutional Review Board of Kaohsiung Medical University Hospital reviewed and approved the study protocol (KMUHIRB-E(I)-20,210,306). The Institutional Review Board of Kaohsiung Medical University Hospital agreed to our request that informed consent be waived because of the use of secondary data analysis with anonymous personal identification numbers. All the study procedures were conducted according to the principles of the Declaration of Helsinki.

### Air pollutants

**Figure 4:**
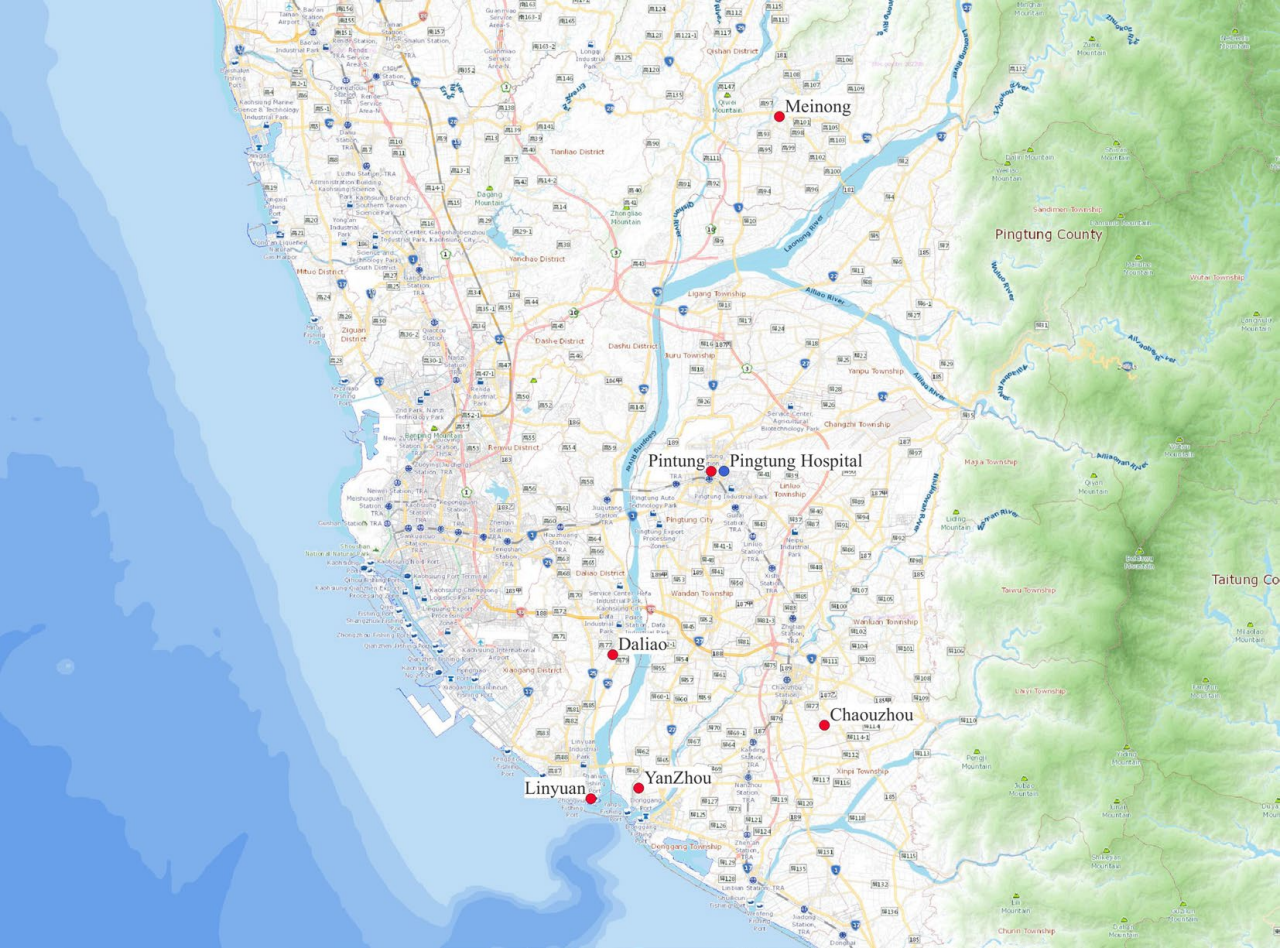
The location of the study hospital and the air quality monitoring stations. Air pollutant data were obtained from five central (Pingtung, Daliao, Linyuan, Meinong, and Chaouzhou) air quality monitoring stations maintained by the Environmental Protection Administration of Taiwan and one local (YanZhou) monitoring station maintained by the Environmental Protection Bureau, Pingtung County. The data from the central stations can be downloaded via the following hyperlink https://airtw.epa.gov.tw/CHT/Query/His_Data.aspx, and the data from the YanZhou station can be downloaded from https://www.ptepb.gov.tw/News2.aspx?n=0CC4FC82AA4C17A8&sms=7130494E45CEF0D8.

The study obtained air pollutant concentrations from 2011 to 2015, including CO, NO, NO_2_, NO_x_, O_3_, PM_2.5_, PM_10_, and SO_2_ from five central (Pingtung, Daliao, Linyuan, Meinong, and Chaouzhou) and one local (YanZhou) air quality monitoring stations near Pingtung Hospital (Fig. 4). In addition, the ambient temperatures were collected as a covariate. The Environmental Protection Administrative of Taiwan and the Environmental Protection Bureau, Pingtung County regularly maintain the stations. The hourly air pollutant data recorded by the central stations were publicly available on websites, while the local station offered daily data through average hourly data^32^.

### Kidney function measurements

Serum creatinine, urine creatinine, and urine protein were measured using the Jaff method with a Beckman Coulter DxC 700 AU Clinical Chemistry analyzer. The patients’ serum creatinine values were obtained from care program enrollment to the end of June 2021 or before withdrawal from the program due to death, dialysis, kidney transplantation, or lost follow-ups. Kidney function was represented by an eGFR that was calculated through the age, sex, and serum creatinine values using a CKD-EPI equation^33^. Since every study subject had several eGFR measurements after the care program enrollment, the eGFR measurements over time were repeated and correlated.

### Covariates

Several baseline covariates, including demographic features (age, sex, educational level, smoking, and alcohol consumption), comorbid conditions (diabetes mellitus, hypertension, cerebrovascular accident, congestive heart failure, ischemic heart disease, gout, and cancer), laboratory data (blood creatinine, urine protein, and urine creatinine), as well as medications with refillable prescriptions for more than 3 months (non-steroidal anti-inflammatory drugs (NSAIDs) and ACEI/ARB) were also recorded. Patients’ demographic characteristics and comorbid conditions were obtained from the records for reimbursement of the pre-ESRD care program. Educational level was classified into three categories (0, 1–12, and > 12 years). Comorbidities were self-reported and confirmed by clinical diagnosis by a trained nurse educator. The medication history was obtained by reviewing electronic medical records, whereby the user was defined by a cumulative prescription of over 3 months during the observed period. In addition, the ambient temperature was used as a covariate because it may confound the effect of air pollutants on health.

### Statistical analysis

The distribution of the patient characteristics was described by mean and standard deviation for continuous variables with approximately normal distribution and median and interquartile range for those with no normal distribution. Differences between air pollutant concentration quartile groups were assessed by one-way ANOVA or the Kruskal–Wallis test. In addition, we applied counts and percentages for the categorical variables of patient characteristics and tested the differences in distributions between groups by Chi-square test or Fisher exact test. The raw air pollutants and ambient temperature data at the central air quality monitoring stations were represented by an hour on each measured date. We first averaged the hourly data, which served as daily concentrations for the central stations. Subsequently, we averaged the daily concentrations of each station during the observed period to represent long-term air pollutant concentrations and ambient temperature for each air quality monitoring station.

Distances were calculated by longitude and latitude to understand the distance between patient residences and air quality monitoring stations. Each patient’s long-term air pollutant concentration was estimated by employing inverse distance weighting with a distance power of 1 approach and the subjects were grouped by different percentiles of each selected air pollutant concentration. First, we examined the potential confounding factors by inspecting the differences in characteristic distributions between each air pollutant concentration quartile. Then, to explore relationships between long-term eGFR changes at different quartiles of air pollutant concentrations, longitudinal spline curves of eGFR change were constructed by cubic splines by setting 50 smoothness. The subjects’ annual eGFR slope was estimated by one mixed model with random intercept and slope. In modelings, patients’ air pollution concentration was grouped by 3 (10th, 50th, and 90th percentile) and 5 knots (5th, 27.5th, 50th, 72.5th, and 95th percentile) as suggested by previous literature^34^ to explore the linear and non-linear relationships of air pollutant concentration with annual eGFR slope.

The univariable and multivariable general linear models were applied to explore the associations between each selected air pollutant concentration and annual eGFR slope. Linear and non-linear relationships were evaluated by putting knot grouping air pollution concentrations and air pollution concentrations with a restricted cubic spline function into models. The optimal fitted number of knots for modeling was evaluated based on the AIC (smaller is better). Log transformation was performed for UPCR due to a highly right-skewed distribution. We first developed model 1 by only putting a group of air pollutant concentrations. Then, model 2 further added age, sex, educational level, diabetes mellitus, hypertension, cerebrovascular accident, congestive heart failure, ischemic heart disease, gout, angiotensin-converting enzyme inhibitor/ angiotensin II receptor blocker, temperature, and log (urine protein creatinine ratio). Finally, the univariable and multivariable analyses were further conducted after transforming air pollutant concentrations through restricted cubic splines. The model results were displayed by regression coefficient and 95% confidence interval (CI), with a two-tailed *p*-value of < 0.05 considered statistically significant. To reflect the complex modeling results, combining trajectory plots and scatter plots were generated for the effects of air pollutants on the annual eGFR slope. All analyses were performed using SAS (version 9.4; SAS Institute Inc., Cary, NC, USA).

### Prior presentation

Some of the study results were presented in abstract and poster form at the December 11–12, 2022, Annual Meeting of the Taiwan Society of Nephrology at the very beginning.

## Supplementary Information

Supplementary Information.

## Data Availability

The data that support the findings of this study are available from the Division of Medical Statistics and Bioinformatics, Department of Medical Research, Kaohsiung Medical University Hospital, Kaohsiung Medical University but restrictions apply to the availability of these data, which were used under license for the current study, and so are not publicly available. Data are however available from the authors upon reasonable request and with permission of the Division of Medical Statistics and Bioinformatics, Department of Medical Research, Kaohsiung Medical University Hospital, Kaohsiung Medical University.
