# Supplementary material for: Associations of air pollutant concentrations with longitudinal kidney function changes in patients with chronic kidney disease
Source: Sci Rep. 2023 Jun 13;13:9609. doi: 10.1038/s41598-023-36682-4 (PMC10264407; doi:10.1038/s41598-023-36682-4)

**Table S1.** Distribution of patient characteristics in the different quartiles of CO concentration.

|  | CO, ppm | | | |  |
| --- | --- | --- | --- | --- | --- |
|  | Q1  (<0.44) | Q2  (0.44-0.46) | Q3  (0.47-0.48) | Q4  (>0.48) | P-value |
| n | 111 | 112 | 112 | 112 |  |
| Age, yrs | 75.05±12.11 | 77.51±12.26 | 76.8±12.17 | 79.83±12.03 | 0.03 |
| Gender |  |  |  |  | 0.36 |
| Men | 62 (55.9) | 63 (56.3) | 73 (65.2) | 71 (63.4) |  |
| Women | 49 (44.1) | 49 (43.8) | 39 (34.8) | 41 (36.6) |  |
| Educational level, yrs (missing n=2) |  |  |  |  | <0.001 |
| 0 | 57 (51.4) | 51 (46) | 49 (43.8) | 34 (30.6) |  |
| 1-12 | 33 (29.7) | 40 (36) | 25 (22.3) | 33 (29.7) |  |
| >12 | 21 (18.9) | 20 (18) | 38 (33.9) | 44 (39.6) |  |
| Smoking | 7 (6.3) | 6 (5.4) | 12 (10.7) | 6 (5.4) | 0.61 |
| Alcohol consumption | 7 (6.3) | 7 (6.3) | 9 (8) | 4 (3.6) | 0.37* |
| Drugs (missing n=36) |  |  |  |  |  |
| NSAIDs | 4 (3.8) | 4 (3.9) | 5 (4.9) | 0 (0) | 0.14* |
| ACEI/ARB | 51 (48.6) | 56 (54.4) | 49 (47.6) | 59 (59) | 0.32 |
| Co-morbidity |  |  |  |  |  |
| Diabetes mellitus | 56 (50.5) | 48 (42.9) | 53 (47.3) | 54 (48.2) | 0.71 |
| Hypertension | 93 (83.8) | 74 (66.1) | 84 (75) | 85 (75.9) | 0.02 |
| Cerebrovascular accident | 13 (11.7) | 9 (8) | 8 (7.1) | 10 (8.9) | 0.66 |
| Congestive heart failure | 11 (9.9) | 7 (6.3) | 7 (6.3) | 13 (11.6) | 0.37 |
| Ischemia heart disease | 11 (9.9) | 10 (8.9) | 7 (6.3) | 4 (3.6) | 0.25 |
| Gout | 14 (12.6) | 8 (7.1) | 12 (10.7) | 12 (10.7) | 0.6 |
| Cancer | 4 (3.6) | 5 (4.5) | 6 (5.4) | 2 (1.8) | 0.58* |
| eGFR at enrollment, ml/min/1.73m^2^ | 30.12±16.2 | 29.44±15.38 | 28.74±14.43 | 29.65±12.54 | 0.92 |
| ≥45 | 13 (11.7) | 6 (5.4) | 5 (4.5) | 9 (8) | 0.65 |
| 30–45 | 43 (38.7) | 45 (40.2) | 48 (42.9) | 47 (42) |  |
| 15–29 | 36 (32.4) | 45 (40.2) | 42 (37.5) | 42 (37.5) |  |
| <15 | 19 (17.1) | 16 (14.3) | 17 (15.2) | 14 (12.5) |  |
| Urine protein creatine ratio, mg/g | 1020  (274-2115) | 688  (236-1850) | 774  (259-1613) | 623  (151-1405) | 0.11^#^ |
| <150 | 17 (15.3) | 17 (15.2) | 10 (8.9) | 30 (26.8) | 0.008 |
| 150–1,000 | 37 (33.3) | 49 (43.8) | 54 (48.2) | 42 (37.5) |  |
| 1,000–2,999 | 34 (30.6) | 34 (30.4) | 35 (31.3) | 30 (26.8) |  |
| >3,000 | 23 (20.7) | 12 (10.7) | 13 (11.6) | 10 (8.9) |  |

Abbreviations: CO, carbon monoxide; ppm, parts per million; Q, quartile; eGFR, estimated glomerular filtration rate; NSAIDs, non-steroidal anti-inflammatory drugs; ACEI/ARB, angiotensin converting enzyme inhibitor/ angiotensin Ⅱ receptor blocker.

Note: The “^*^” indicates the p values generated by the Fisher exact test because at least 25% of cells' expected number is less than 5. The “^#^” shows the p values generated by the Kruskal-Wallis Test.

**Table S2.** Distribution of patient characteristics in the different quartiles of NO concentration.

|  | NO, ppb | | | |  |
| --- | --- | --- | --- | --- | --- |
|  | Q1  (<2.39) | Q2  (2.39-2.41) | Q3  (2.42-2.43) | Q4  (>2.43) | P-value |
| n | 111 | 112 | 112 | 112 |  |
| Age, yrs | 79.4±11.95 | 76±12.71 | 77.24±12.42 | 76.59±11.68 | 0.18 |
| Gender |  |  |  |  | 0.83 |
| Men | 69 (62.2) | 70 (62.5) | 65 (58.0) | 65 (58.0) |  |
| Women | 42 (37.8) | 42 (37.5) | 47 (42.0) | 47 (42.0) |  |
| Educational level, yrs (missing n=2) |  |  |  |  | 0.43 |
| 0 | 44 (40.0) | 42 (37.5) | 52 (46.4) | 53 (47.8) |  |
| 1-12 | 32 (29.1) | 32 (28.6) | 33 (29.5) | 34 (30.6) |  |
| >12 | 34 (30.9) | 38 (33.9) | 27 (24.1) | 24 (21.6) |  |
| Smoking | 6 (5.4) | 11 (9.8) | 7 (6.3) | 7 (6.3) | 0.86 |
| Alcohol consumption | 7 (6.3) | 4 (3.6) | 7 (6.3) | 9 (8.0) | 0.64* |
| Drugs (missing n=36) |  |  |  |  |  |
| NSAIDs | 2 (2.0) | 3 (2.8) | 4 (4.0) | 4 (3.9) | 0.88* |
| ACEI/ARB | 51 (51.0) | 58 (54.7) | 47 (46.5) | 59 (56.7) | 0.48 |
| Co-morbidity |  |  |  |  |  |
| Diabetes mellitus | 54 (48.7) | 55 (49.1) | 49 (43.8) | 53 (47.3) | 0.85 |
| Hypertension | 83 (74.8) | 93 (83) | 86 (76.8) | 74 (66.1) | 0.03 |
| Cerebrovascular accident | 13 (11.7) | 6 (5.4) | 12 (10.7) | 9 (8) | 0.34 |
| Congestive heart failure | 12 (10.8) | 9 (8.0) | 9 (8.0) | 8 (7.1) | 0.78 |
| Ischemia heart disease | 6 (5.4) | 6 (5.4) | 9 (8.0) | 11 (9.8) | 0.50 |
| Gout | 13 (11.7) | 9 (8.0) | 14 (12.5) | 10 (8.9) | 0.64 |
| Cancer | 2 (1.8) | 8 (7.1) | 2 (1.8) | 5 (4.5) | 0.14* |
| eGFR at enrollment, ml/min/1.73m^2^ | 31.36±14.95 | 28.1±13.01 | 28.78±15.28 | 29.73±15.26 | 0.38 |
| ≥45 | 11 (9.9) | 7 (6.2) | 7 (6.2) | 8 (7.1) | 0.51 |
| 30–45 | 49 (44.1) | 48 (42.9) | 41 (36.6) | 45 (40.2) |  |
| 15–29 | 40 (36.1) | 35 (31.3) | 45 (40.2) | 45 (40.2) |  |
| <15 | 11 (9.9) | 22 (19.6) | 19 (17.0) | 14 (12.5) |  |
| Urine protein creatine ratio, mg/g | 776  (204-1759) | 564  (191-1629) | 833  (255-1887) | 800  (273-1805) | 0.5 |
| <150 | 22 (19.8) | 24 (21.4) | 13 (11.6) | 15 (13.4) | 0.58 |
| 150–1,000 | 42 (37.8) | 45 (40.2) | 48 (42.9) | 47 (42.0) |  |
| 1,000–2,999 | 33 (29.7) | 29 (25.9) | 33 (29.5) | 38 (33.9) |  |
| >3,000 | 14 (12.6) | 14 (12.5) | 18 (16.1) | 12 (10.7) |  |

Abbreviations: NO, nitrogen monoxide; ppb, parts per billion; Q, quartile; eGFR, estimated glomerular filtration rate; NSAIDs, non-steroidal anti-inflammatory drugs; ACEI/ARB, angiotensin converting enzyme inhibitor/ angiotensin Ⅱ receptor blocker.

Note: The “^*^” indicates the p values generated by the Fisher exact test because at least 25% of cells' expected number is less than 5. The “^#^” shows the p values generated by the Kruskal-Wallis Test.

**Table S3.** Distribution of patient characteristics in the different quartiles of NO_2_ concentration.

|  | NO_2_, ppb | | | |  |
| --- | --- | --- | --- | --- | --- |
|  | Q1  (<12.93) | Q2  (12.93-13.65) | Q3  (13.66-13.90) | Q4  (>13.90) | P-value |
| n | 111 | 112 | 112 | 112 |  |
| Age, yrs | 75.22±12.51 | 77.67±12.37 | 76.93±12.01 | 79.38±11.8 | 0.08 |
| Gender |  |  |  |  | 0.32 |
| Men | 60 (54.0) | 66 (58.9) | 74 (66.1) | 69 (61.6) |  |
| Women | 51 (46.0) | 46 (41.1) | 38 (33.9) | 43 (38.4) |  |
| Educational level, yrs (missing n=2) |  |  |  |  | 0.004 |
| <1 | 55 (49.6) | 51 (46.0) | 50 (44.6) | 35 (31.6) |  |
| 1-12 | 36 (32.4) | 37 (33.3) | 25 (22.3) | 33 (29.7) |  |
| >12 | 20 (18.0) | 23 (20.7) | 37 (33.1) | 43 (38.7) |  |
| Smoking | 6 (5.4) | 7 (6.3) | 12 (10.7) | 6 (5.4) | 0.56 |
| Alcohol consumption | 7 (6.3) | 7 (6.3) | 8 (7.1) | 5 (4.5) | 0.53* |
| Drugs (missing n=36) |  |  |  |  |  |
| NSAIDs | 3 (2.9) | 4 (3.8) | 5 (4.9) | 1 (1.0) | 0.48* |
| ACEI/ARB | 48 (47.1) | 61 (58.1) | 47 (45.6) | 59 (58.4) | 0.12 |
| Co-morbidity |  |  |  |  |  |
| Diabetes mellitus | 58 (52.3) | 47 (42.0) | 51 (45.5) | 55 (49.1) | 0.45 |
| Hypertension | 95 (85.6) | 74 (66.1) | 82 (73.2) | 85 (75.9) | 0.009 |
| Cerebrovascular accident | 13 (11.7) | 11 (9.8) | 5 (4.5) | 11 (9.8) | 0.26 |
| Congestive heart failure | 10 (9.0) | 10 (8.9) | 7 (6.3) | 11 (9.8) | 0.79 |
| Ischemia heart disease | 9 (8.1) | 12 (10.7) | 6 (5.4) | 5 (4.5) | 0.26 |
| Gout | 13 (11.7) | 9 (8.0) | 12 (10.7) | 12 (10.7) | 0.82 |
| Cancer | 5 (4.5) | 3 (2.7) | 7 (6.3) | 2 (1.8) | 0.32* |
| eGFR at enrollment, ml/min/1.73m^2^ | 29.96±16.6 | 29.5±14.79 | 28.58±15.27 | 29.9±11.69 | 0.89 |
| ≥45 | 13 (11.7) | 6 (5.4) | 6 (5.4) | 8 (7.2) | 0.49 |
| 30–45 | 42 (37.8) | 45 (40.2) | 46 (41.0) | 50 (44.6) |  |
| 15–29 | 36 (32.5) | 47 (42.0) | 41 (36.6) | 41 (36.6) |  |
| <15 | 20 (18.0) | 14 (12.4) | 19 (17.0) | 13 (11.6) |  |
| Urine protein creatine ratio, mg/g | 1082  (282-2770) | 667  (235-1380) | 783  (259-1764) | 615  (153-1351) | 0.01 |
| <150 | 17 (15.4) | 17 (15.2) | 10 (8.9) | 30 (26.8) | 0.001 |
| 150–1,000 | 36 (32.4) | 53 (47.3) | 50 (44.6) | 43 (38.4) |  |
| 1,000–2,999 | 33 (29.7) | 34 (30.4) | 37 (33.1) | 29 (25.9) |  |
| >3,000 | 25 (22.5) | 8 (7.1) | 15 (13.4) | 10 (8.9) |  |

Abbreviations: NO_2_, nitrogen dioxide; ppb, parts per billion; Q, quartile; eGFR, estimated glomerular filtration rate; NSAIDs, non-steroidal anti-inflammatory drugs; ACEI/ARB, angiotensin converting enzyme inhibitor/ angiotensin Ⅱ receptor blocker.

Note: The “^*^” indicates the p values generated by the Fisher exact test because at least 25% of cells' expected number is less than 5. The “^#^” shows the p values generated by the Kruskal-Wallis Test.

**Table S4.** Distribution of characteristics in the different quartiles of NO_x_ concentration.

|  | NO_x_, ppb | | | |  |
| --- | --- | --- | --- | --- | --- |
|  | Q1  (<15.34) | Q2  (15.34-16.07) | Q3  (16.08-16.28) | Q4  (>16.28) | P-value |
| n | 111 | 112 | 112 | 112 |  |
| Age, yrs | 75.23±12.67 | 78.55±12 | 76.09±12.11 | 79.31±11.8 | 0.04 |
| Gender |  |  |  |  | 0.60 |
| Men | 61 (55.0) | 69 (61.6) | 68 (60.7) | 71 (63.4) |  |
| Women | 50 (45.0) | 43 (38.4) | 44 (39.3) | 41 (36.6) |  |
| Educational level, yrs (missing n=2) |  |  |  |  | 0.03 |
| 0 | 54 (48.7) | 49 (43.8) | 52 (46.9) | 36 (32.5) |  |
| 1-12 | 36 (32.4) | 36 (32.1) | 27 (24.3) | 32 (28.8) |  |
| >12 | 21 (18.9) | 27 (24.1) | 32 (28.8) | 43 (38.7) |  |
| Smoking | 6 (5.4) | 6 (5.4) | 13 (11.6) | 6 (5.4) | 0.45 |
| Alcohol consumption | 7 (6.3) | 6 (5.4) | 9 (8.0) | 5 (4.5) | 0.46* |
| Drugs (missing n=36) |  |  |  |  |  |
| NSAID | 3 (2.9) | 5 (4.8) | 4 (3.9) | 1 (1.0) | 0.50* |
| ACEI/ARB | 48 (46.6) | 54 (51.9) | 54 (52.4) | 59 (58.4) | 0.41 |
| Co-morbidity |  |  |  |  |  |
| Diabetes mellitus | 59 (53.2) | 46 (41.1) | 52 (46.4) | 54 (48.2) | 0.34 |
| Hypertension | 94 (84.7) | 76 (67.9) | 83 (74.1) | 83 (74.1) | 0.03 |
| Cerebrovascular accident | 13 (11.7) | 10 (8.9) | 6 (5.4) | 11 (9.8) | 0.41 |
| Congestive heart failure | 10 (9.0) | 11 (9.8) | 6 (5.4) | 11 (9.8) | 0.58 |
| Ischemia heart disease | 10 (9.0) | 9 (8.0) | 8 (7.1) | 5 (4.5) | 0.59 |
| Gout | 14 (12.6) | 9 (8.0) | 10 (8.9) | 13 (11.6) | 0.64 |
| Cancer | 5 (4.5) | 3 (2.7) | 7 (6.3) | 2 (1.8) | 0.32* |
| eGFR at enrollment, ml/min/1.73m^2^ | 30.21±16.5 | 30±15.56 | 27.68±14.54 | 30.06±11.62 | 0.52 |
| ≥45 | 13 (11.7) | 7 (6.3) | 5 (4.5) | 8 (7.1) | 0.39 |
| 30–45 | 43 (38.7) | 44 (39.3) | 46 (41.1) | 50 (44.6) |  |
| 15–29 | 36 (32.4) | 47 (42.0) | 40 (35.7) | 42 (37.5) |  |
| <15 | 19 (17.1) | 14 (12.5) | 21 (18.8) | 12 (10.7) |  |
| Urine protein creatine ratio, mg/g | 1082  (282-2770) | 575  (215-1218) | 938  (284-1805) | 615  (153-1405) | 0.02^#^ |
| <150 | 17 (15.3) | 20 (17.9) | 7 (6.3) | 30 (26.8) | <0.001 |
| 150–1,000 | 36 (32.4) | 53 (47.3) | 50 (44.6) | 43 (38.4) |  |
| 1,000–2,999 | 33 (29.7) | 32 (28.6) | 39 (34.8) | 29 (25.9) |  |
| >3,000 | 25 (22.6) | 7 (6.3) | 16 (14.3) | 10 (8.9) |  |

Abbreviations: ppb, parts per billion; Q, quartile; NO_x_, nitrogen oxides; eGFR, estimated glomerular filtration rate; NSAIDs, non-steroidal anti-inflammatory drugs; ACEI/ARB, angiotensin converting enzyme inhibitor/ angiotensin Ⅱ receptor blocker.

Note: The “^*^” indicates the p values generated by the Fisher exact test because at least 25% of cells' expected number is less than 5. The “^#^” shows the p values generated by the Kruskal-Wallis Test.

**Table S5.** Distribution of characteristics in the different quartiles of O_3_ concentration.

|  | O_3_ ppb | | | |  |
| --- | --- | --- | --- | --- | --- |
|  | Q1  (<31.30) | Q2  (31.30-31.51) | Q3  (31.52-31.71) | Q4  (>31.71) | P-value |
| n | 111 | 112 | 112 | 112 |  |
| Age, yrs | 75.5 ±12.64 | 77.14 ±11.57 | 77.04 ±12.15 | 79.5 ±12.34 | 0.11 |
| Gender |  |  |  |  | 0.18 |
| Men | 58 (52.2) | 66 (58.9) | 73 (65.2) | 72 (64.3) |  |
| Women | 53 (47.8) | 46 (41.1) | 39 (34.8) | 40 (35.7) |  |
| Educational level, yrs (missing n=2) |  |  |  |  | 0.002 |
| 0 | 55 (50.0) | 52 (46.4) | 50 (44.6) | 34 (30.6) |  |
| 1-12 | 33 (30.0) | 39 (34.8) | 25 (22.3) | 34 (30.6) |  |
| >12 | 22 (20.0) | 21 (18.8) | 37 (33.1) | 43 (38.8) |  |
| Smoking | 7 (6.3) | 6 (5.4) | 11 (9.8) | 7 (6.3) | 0.81 |
| Alcohol consumption | 8 (7.2) | 6 (5.4) | 9 (8) | 4 (3.6) | 0.34^*^ |
| Drugs (missing n=36) |  |  |  |  |  |
| NSAIDs | 4 (3.9) | 4 (3.7) | 5 (5.0) | 0 (0.0) | 0.14^*^ |
| ACEI/ARB | 58 (56.3) | 53 (49.5) | 47 (46.5) | 57 (57) | 0.36 |
| Co-morbidity |  |  |  |  |  |
| Diabetes mellitus | 57 (51.4) | 49 (43.8) | 52 (46.4) | 53 (47.3) | 0.72 |
| Hypertension | 85 (76.6) | 84 (75) | 83 (74.1) | 84 (75) | 0.78 |
| Cerebrovascular accident | 12 (10.8) | 8 (7.1) | 10 (8.9) | 10 (8.9) | 0.82 |
| Congestive heart failure | 9 (8.1) | 10 (8.9) | 6 (5.4) | 13 (11.6) | 0.41 |
| Ischemia heart disease | 11 (9.9) | 10 (8.9) | 7 (6.3) | 4 (3.6) | 0.06 |
| Gout | 11 (9.9) | 12 (10.7) | 11 (9.8) | 12 (10.7) | 0.99 |
| Cancer | 1 (0.9) | 8 (7.1) | 5 (4.5) | 3 (2.7) | 0.09^*^ |
| eGFR at enrollment, ml/min/1.73m^2^ | 31.00±15.28 | 28.87 ±16.03 | 28.88 ±14.73 | 29.20 ±12.43 | 0.66 |
| ≥45 | 11 (9.9) | 8 (7.1) | 6 (5.3) | 8 (7.1) | 0.85 |
| 30–45 | 48 (43.3) | 40 (35.7) | 47 (42.0) | 48 (42.9) |  |
| 15–29 | 39 (35.1) | 43 (38.4) | 43 (38.4) | 40 (35.7) |  |
| <15 | 13 (11.7) | 21 (18.8) | 16 (14.3) | 16 (14.3) |  |
| Urine protein creatine ratio, mg/g | 863  (247-1772) | 689  (265-1985) | 794  (259-1617) | 623  (159-1405) | 0.28^#^ |
| <150 | 19 (17.1) | 15 (13.3) | 11 (9.8) | 29 (25.9) | 0.07 |
| 150–1,000 | 41 (36.9) | 46 (41.1) | 53 (47.3) | 42 (37.5) |  |
| 1,000–2,999 | 36 (32.5) | 31 (27.7) | 36 (32.2) | 30 (26.8) |  |
| >3,000 | 15 (13.5) | 20 (17.9) | 12 (10.7) | 11 (9.8) |  |

Abbreviations: O_3_, ozone; ppb, parts per billion; Q, quartile; eGFR, estimated glomerular filtration rate; NSAIDs, non-steroidal anti-inflammatory drugs; ACEI/ARB, angiotensin converting enzyme inhibitor/ angiotensin Ⅱ receptor blocker.

Note: The “^*^” indicates the p values generated by the Fisher exact test because at least 25% of cells' expected number is less than 5. The “^#^” shows the p values generated by the Kruskal-Wallis Test.

**Table S6.** Distribution of characteristics in the different quartiles of PM_10_ concentration.

|  | PM_10,_ μg/m^3^ | | | |  |
| --- | --- | --- | --- | --- | --- |
|  | Q1  (<63.42) | Q2  (63.42-64.65) | Q3  (64.66-65.18) | Q4  (>65.18) | P-value |
| n | 111 | 112 | 112 | 112 |  |
| Age, yrs | 75.18±12.04 | 77.19±12.44 | 76.99±12.09 | 79.83±12.03 | 0.04 |
| Gender |  |  |  |  | 0.26 |
| Men | 59 (53.1) | 66 (58.9) | 73 (65.2) | 71 (63.4) |  |
| Women | 52 (46.9) | 46 (41.1) | 39 (34.8) | 41 (36.6) |  |
| Educational level, yrs (missing n=2) |  |  |  |  | 0.001 |
| <1 | 56 (50.5) | 50 (45.1) | 51 (45.5) | 34 (30.6) |  |
| 1-12 | 35 (31.5) | 39 (35.1) | 24 (21.4) | 33 (29.8) |  |
| >12 | 20 (18.0) | 22 (19.8) | 37 (33.1) | 44 (39.6) |  |
| Smoking | 6 (5.4) | 8 (7.1) | 11 (9.8) | 6 (5.4) | 0.79 |
| Alcohol consumption | 7 (6.3) | 7 (6.3) | 9 (8.0) | 4 (3.6) | 0.37^*^ |
| Drugs (missing n=36) |  |  |  |  |  |
| NSAIDs | 3 (2.9) | 5 (4.8) | 5 (4.8) | 0 (0.0) | 0.11^*^ |
| ACEI/ARB | 51 (50.0) | 56 (53.3) | 49 (47.1) | 59 (59.0) | 0.37 |
| Co-morbidity |  |  |  |  |  |
| Diabetes mellitus | 59 (53.2) | 46 (41.1) | 52 (46.4) | 54 (48.2) | 0.34 |
| Hypertension | 93 (83.8) | 75 (67.0) | 83 (74.1) | 85 (75.9) | 0.04 |
| Cerebrovascular accident | 13 (11.7) | 10 (8.9) | 7 (6.3) | 10 (8.9) | 0.56 |
| Congestive heart failure | 10 (9.0) | 9 (8.0) | 6 (5.4) | 13 (11.6) | 0.41 |
| Ischemia heart disease | 9 (8.1) | 11 (9.8) | 8 (7.1) | 4 (3.6) | 0.32 |
| Gout | 12 (10.8) | 10 (8.9) | 12 (10.7) | 12 (10.7) | 0.96 |
| Cancer | 4 (3.6) | 4 (3.6) | 7 (6.3) | 2 (1.8) | 0.42^*^ |
| eGFR at enrollment, ml/min/1.73m^2^ | 30.69±16.46 | 29.12±14.93 | 28.5±14.54 | 29.65±12.54 | 0.72 |
| ≥45 | 14 (12.6) | 5 (4.4) | 5 (4.4) | 9 (8.0) | 0.35 |
| 30–45 | 44 (39.6) | 44 (39.3) | 48 (42.9) | 47 (42.0) |  |
| 15–29 | 35 (31.6) | 48 (42.9) | 40 (35.7) | 42 (37.5) |  |
| <15 | 18 (16.2) | 15 (13.4) | 19 (17.0) | 14 (12.5) |  |
| Urine protein creatine ratio, mg/g | 1124  (282-2632) | 671  (239-1549) | 757  (258-1617) | 623  (151-1405) | 0.04^#^ |
| <150 | 17 (15.3) | 17 (15.2) | 10 (8.9) | 30 (26.8) | 0.003 |
| 150–1,000 | 35 (31.5) | 51 (45.5) | 54 (48.2) | 42 (37.5) |  |
| 1,000–2,999 | 35 (31.5) | 33 (29.5) | 35 (31.3) | 30 (26.8) |  |
| >3,000 | 24 (21.6) | 11 (9.8) | 13 (11.6) | 10 (8.9) |  |

Abbreviations: PM_10_, particulate matter <10 μm in aerodynamic diameter; μg/m^3^, micrograms per cubic meter; Q, quartile; eGFR, estimated glomerular filtration rate; NSAIDs, non-steroidal anti-inflammatory drugs; ACEI/ARB, angiotensin converting enzyme inhibitor/ angiotensin Ⅱ receptor blocker.

Note: The “^*^” indicates the p values generated by the Fisher exact test because at least 25% of cells' expected number is less than 5. The “^#^” shows the p values generated by the Kruskal-Wallis Test.

**Table S7.** Distribution of characteristics in the different quartiles of PM_2.5_ concentration.

|  | PM_2.5,_ μg/m^3^ | | | |  | |
| --- | --- | --- | --- | --- | --- | --- |
|  | Q1  (<35.00) | Q2  (35.00-36.10) | Q3  (36.11-36.46) | Q4  (>36.46) | | P-value |
| n | 111 | 112 | 112 | 112 | |  |
| Age, yrs | 76.03±11.95 | 76.54±12.53 | 76.79±12.17 | 79.83±12.03 | | 0.09 |
| Gender |  |  |  |  | | 0.21 |
| Men | 58 (52.2) | 67 (59.8) | 73 (65.2) | 71 (63.4) | |  |
| Women | 53 (47.8) | 45 (40.2) | 39 (34.8) | 41 (36.6) | |  |
| Educational level, yrs (missing n=2) |  |  |  |  | | 0.001 |
| 0 | 56 (50.5) | 51 (46.0) | 50 (44.6) | 34 (30.6) | |  |
| 1-12 | 34 (30.6) | 39 (35.1) | 25 (22.4) | 33 (29.8) | |  |
| >12 | 21 (18.9) | 21 (18.9) | 37 (33.0) | 44 (39.6) | |  |
| Smoking | 5 (4.5) | 8 (7.1) | 12 (10.7) | 6 (5.4) | | 0.57 |
| Alcohol consumption | 8 (7.2) | 5 (4.5) | 10 (8.9) | 4 (3.6) | | 0.3^*^ |
| Drugs (missing n=36) |  |  |  |  | |  |
| NSAIDs | 4 (3.9) | 4 (3.9) | 5 (4.8) | 0 (0.0) | | 0.13^*^ |
| ACEI/ARB | 52 (50.5) | 55 (52.9) | 49 (47.1) | 59 (59) | | 0.38 |
| Co-morbidity |  |  |  |  | |  |
| Diabetes mellitus | 57 (51.4) | 47 (42) | 53 (47.3) | 54 (48.2) | | 0.56 |
| Hypertension | 93 (83.8) | 74 (66.1) | 84 (75.0) | 85 (75.9) | | 0.02 |
| Cerebrovascular accident | 13 (11.7) | 9 (8.0) | 8 (7.1) | 10 (8.9) | | 0.66 |
| Congestive heart failure | 11 (9.9) | 7 (6.3) | 7 (6.3) | 13 (11.6) | | 0.37 |
| Ischemia heart disease | 10 (9) | 11 (9.8) | 7 (6.3) | 4 (3.6) | | 0.25 |
| Gout | 14 (12.6) | 8 (7.1) | 12 (10.7) | 12 (10.7) | | 0.6 |
| Cancer | 4 (3.6) | 5 (4.5) | 6 (5.4) | 2 (1.8) | | 0.58^*^ |
| eGFR at enrollment, ml/min/1.73m^2^ | 30.23±16.1 | 29.36±15.5 | 28.71±14.41 | 29.65±12.54 | | 0.89 |
| ≥45 | 12 (10.8) | 7 (6.3) | 5 (4.5) | 9 (8.0) | | 0.88 |
| 30–45 | 44 (39.6) | 44 (39.3) | 48 (42.9) | 47 (42.0) | |  |
| 15–29 | 38 (34.2) | 43 (38.4) | 42 (37.5) | 42 (37.5) | |  |
| <15 | 17 (15.3) | 18 (16.0) | 17 (15.2) | 14 (12.5) | |  |
| Urine protein creatine ratio, mg/g | 982  (274-2115) | 790  (260-1811) | 757  (258-1613) | 623  (151-1405) | | 0.13^#^ |
| <150 | 18 (16.2) | 16 (14.3) | 10 (8.9) | 30 (26.8) | | 0.008 |
| 150–1,000 | 38 (34.2) | 48 (42.9) | 54 (48.2) | 42 (37.5) | |  |
| 1,000–2,999 | 32 (28.8) | 36 (32.1) | 35 (31.3) | 30 (26.8) | |  |
| >3,000 | 23 (20.7) | 12 (10.7) | 13 (11.6) | 10 (8.9) | |  |

Abbreviations: PM_2.5_, particulate matter <2.5 μm in aerodynamic diameter; μg/m^3^, micrograms per cubic meter; Q, quartile; eGFR, estimated glomerular filtration rate; NSAIDs, non-steroidal anti-inflammatory drugs; ACEI/ARB, angiotensin converting enzyme inhibitor/ angiotensin Ⅱ receptor blocker.

Note: The “^*^” indicates the p values generated by the Fisher exact test because at least 25% of cells' expected number is less than 5. The “^#^” shows the p values generated by the Kruskal-Wallis Test.

**Table S8.** Distribution of characteristics in the different quartiles of SO_2_ concentration.

|  | SO_2_, ppb | | | |  |
| --- | --- | --- | --- | --- | --- |
|  | Q1  (<3.51) | Q2  (3.51-3.59) | Q3  (3.60-3.70) | Q4  (>3.70) | P-value |
| n | 111 | 112 | 112 | 112 |  |
| Age, yrs | 79.27±12.06 | 76.27±12.83 | 77.01±11.19 | 76.68±12.68 | 0.26 |
| Gender |  |  |  |  | 0.30 |
| Men | 68 (61.3) | 71 (63.4) | 71 (63.4) | 59 (52.7) |  |
| Women | 43 (38.7) | 41 (36.6) | 41 (36.6) | 53 (47.3) |  |
| Educational level, yrs (missing n=2) |  |  |  |  | 0.004 |
| 0 | 39 (35.5) | 43 (38.4) | 53 (47.3) | 56 (50.5) |  |
| 1-12 | 32 (29.0) | 27 (24.1) | 40 (35.7) | 32 (28.8) |  |
| >12 | 39 (35.5) | 42 (37.5) | 19 (17.0) | 23 (20.7) |  |
| Smoking | 8 (7.2) | 8 (7.1) | 9 (8.0) | 6 (5.4) | 0.93 |
| Alcohol consumption | 6 (5.4) | 5 (4.5) | 10 (8.9) | 6 (5.4) | 0.5^*^ |
| Drugs (missing n=36) |  |  |  |  |  |
| NSAIDs | 1 (1.0) | 3 (2.9) | 4 (3.9) | 5 (4.7) | 0.5^*^ |
| ACEI/ARB | 53 (53.5) | 57 (55.3) | 41 (40.2) | 64 (59.8) | 0.03 |
| Co-morbidity |  |  |  |  |  |
| Diabetes mellitus | 52 (46.9) | 54 (48.2) | 48 (42.9) | 57 (50.9) | 0.68 |
| Hypertension | 84 (75.7) | 90 (80.4) | 82 (73.2) | 80 (71.4) | 0.44 |
| Cerebrovascular accident | 11 (9.9) | 8 (7.1) | 10 (8.9) | 11 (9.8) | 0.88 |
| Congestive heart failure | 12 (10.8) | 6 (5.4) | 12 (10.7) | 8 (7.1) | 0.37 |
| Ischemia heart disease | 4 (3.6) | 6 (5.4) | 8 (7.1) | 14 (12.5) | 0.06 |
| Gout | 13 (11.7) | 11 (9.8) | 11 (9.8) | 11 (9.8) | 0.96 |
| Cancer | 3 (2.7) | 6 (5.4) | 7 (6.3) | 1 (0.9) | 0.12^*^ |
| eGFR at enrollment, ml/min/1.73m^2^ | 30.19±12.67 | 27.21±14.5 | 29.81±14.69 | 30.73±16.44 | 0.28 |
| ≥45 | 9 (8.1) | 7 (6.3) | 7 (6.2) | 10 (8.9) | 0.85 |
| 30–45 | 50 (45.1) | 42 (37.5) | 44 (39.3) | 47 (42.0) |  |
| 15–29 | 39 (35.1) | 41 (36.6) | 45 (40.2) | 40 (35.7) |  |
| <15 | 13 (11.7) | 22 (19.6) | 16 (14.3) | 15 (13.4) |  |
| Urine protein creatine ratio, mg/g | 619  (155-1434) | 957  (251-1772) | 686  (255-1922) | 797  (261-1778) | 0.23^#^ |
| <150 | 30 (27.1) | 16 (14.3) | 10 (8.9) | 18 (16.1) | 0.03 |
| 150–1,000 | 42 (37.8) | 42 (37.5) | 53 (47.3) | 45 (40.2) |  |
| 1,000–2,999 | 28 (25.2) | 38 (33.9) | 30 (26.8) | 37 (33.0) |  |
| >3,000 | 11 (9.9) | 16 (14.2) | 19 (17.0) | 12 (10.7) |  |

Abbreviations: SO_2_, sulfur dioxide; ppb, parts per billion; Q, quartile; eGFR, estimated glomerular filtration rate; NSAIDs, non-steroidal anti-inflammatory drugs; ACEI/ARB, angiotensin converting enzyme inhibitor/ angiotensin Ⅱ receptor blocker.

Note: The “^*^” indicates the p values generated by the Fisher exact test because at least 25% of cells' expected number is less than 5. The “^#^” shows the p values generated by the Kruskal-Wallis Test.

**Table S9**. Checklist of STROBE items of the cohort study.

|  | Item No | Recommendation | Page No |
| --- | --- | --- | --- |
| **Title and abstract** | 1 | (*a*) Indicate the study’s design with a commonly used term in the title or the abstract | 1,2 |
|  |  | (*b*) Provide in the abstract an informative and balanced summary of what was done and what was found | 2 |
| Introduction | | | |
| Background/rationale | 2 | Explain the scientific background and rationale for the investigation being reported | 3 |
| Objectives | 3 | State specific objectives, including any prespecified hypotheses | 4 |
| Methods | | | |
| Study design | 4 | Present key elements of study design early in the paper | 10 |
| Setting | 5 | Describe the setting, locations, and relevant dates, including periods of recruitment, exposure, follow-up, and data collection | 10 |
| Participants | 6 | (*a*) Give the eligibility criteria, and the sources and methods of selection of participants. Describe methods of follow-up | 10 |
|  |  | (*b*) For matched studies, give matching criteria and number of exposed and unexposed |  |
| Variables | 7 | Clearly define all outcomes, exposures, predictors, potential confounders, and effect modifiers. Give diagnostic criteria, if applicable | 11 |
| Data sources/ measurement | 8* | For each variable of interest, give sources of data and details of methods of assessment (measurement). Describe comparability of assessment methods if there is more than one group | 5,6 |
| Bias | 9 | Describe any efforts to address potential sources of bias | 9,12 |
| Study size | 10 | Explain how the study size was arrived at | 10,18 |
| Quantitative variables | 11 | Explain how quantitative variables were handled in the analyses. If applicable, describe which groupings were chosen and why | 11,12 |
| Statistical methods | 12 | (*a*) Describe all statistical methods, including those used to control for confounding | 11,12 |
|  |  | (*b*) Describe any methods used to examine subgroups and interactions | 11,12 |
|  |  | (*c*) Explain how missing data were addressed | 18 |
|  |  | (*d*) If applicable, explain how loss to follow-up was addressed | 18 |
|  |  | (*e*) Describe any sensitivity analyses | 11,12 |
| Results | | |  |
| Participants | 13* | (a) Report numbers of individuals at each stage of study—eg numbers potentially eligible, examined for eligibility, confirmed eligible, included in the study, completing follow-up, and analysed | 5,18 |
|  |  | (b) Give reasons for non-participation at each stage | 5,18 |
|  |  | (c) Consider use of a flow diagram | 18 |
| Descriptive data | 14* | (a) Give characteristics of study participants (eg demographic, clinical, social) and information on exposures and potential confounders | 5,20 |
|  |  | (b) Indicate number of participants with missing data for each variable of interest | 18 |
|  |  | (c) Summarise follow-up time (eg, average and total amount) | 5 |
| Outcome data | 15* | Report numbers of outcome events or summary measures over time | 5,6 |
| Main results | 16 | (*a*) Give unadjusted estimates and, if applicable, confounder-adjusted estimates and their precision (eg, 95% confidence interval). Make clear which confounders were adjusted for and why they were included | 5,6 |
|  |  | (*b*) Report category boundaries when continuous variables were categorized | 5 |
|  |  | (*c*) If relevant, consider translating estimates of relative risk into absolute risk for a meaningful time period |  |
| Other analyses | 17 | Report other analyses done—eg analyses of subgroups and interactions, and sensitivity analyses | 5,6 |
| Discussion | | | |
| Key results | 18 | Summarise key results with reference to study objectives | 7 |
| Limitations | 19 | Discuss limitations of the study, taking into account sources of potential bias or imprecision. Discuss both direction and magnitude of any potential bias | 9 |
| Interpretation | 20 | Give a cautious overall interpretation of results considering objectives, limitations, multiplicity of analyses, results from similar studies, and other relevant evidence | 7,8,9 |
| Generalisability | 21 | Discuss the generalisability (external validity) of the study results | 7,8,9 |
| Other information | | | |
| Funding | 22 | Give the source of funding and the role of the funders for the present study and, if applicable, for the original study on which the present article is based | 16 |

*Give information separately for exposed and unexposed groups.

Figure S1. The number of study patients with estimated glomerular filtration rate measurements in each follow-up year.

Footnote for Figure S1: There are 447 patients, with 5,811 estimated glomerular filtration rate measurements, in the study period. The patient numbers through 0-1, 1-2, 2-3, 3-4, 4-5, 5-6, 6-7, 7-8, 8-9, 9-10, and >10 years are 447, 335, 260, 188, 152, 127, 86, 52, 24, and 12, respectively.


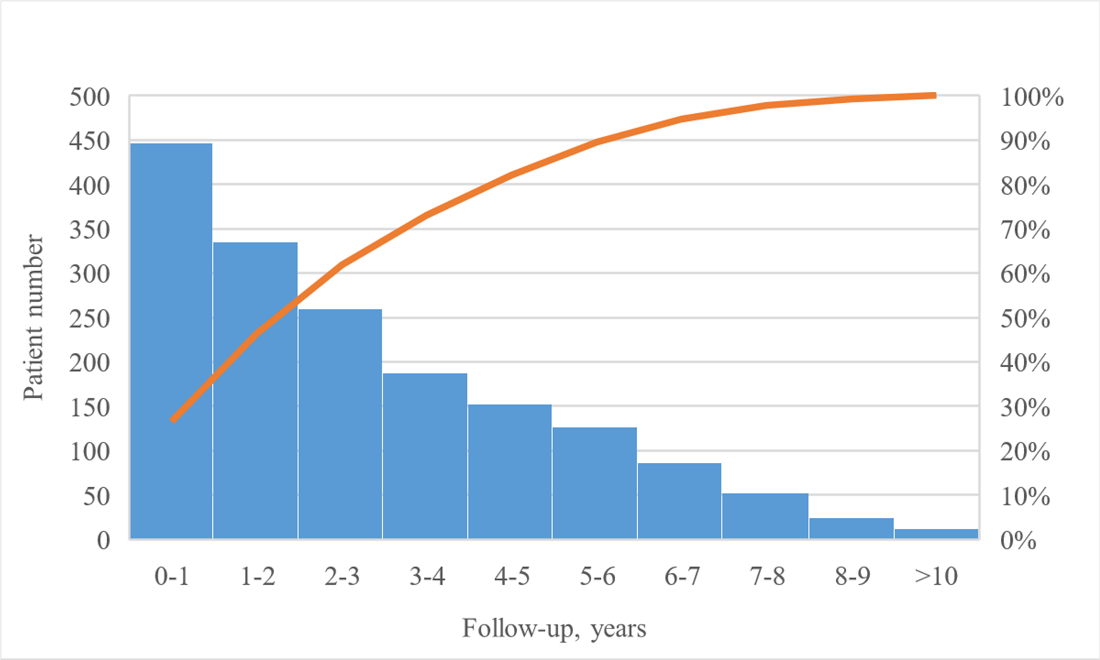


Figure S2. Renal function trajectory in the quartile of ambient air pollutant concentration. A) CO; B) NO; C) NO_2_; D) NO_x_; E) O_3_; F) PM_10_; G) PM_2.5_; H) SO_2_.

Footnote of Figure S4: The plots produced cubic splines by setting 50 smoothness in the spline curves. A value close to zero results in curves with many wiggles, which then nearly pass through every observed value. This value close to 100 results in flat curves that are nearly the least-square regression line.


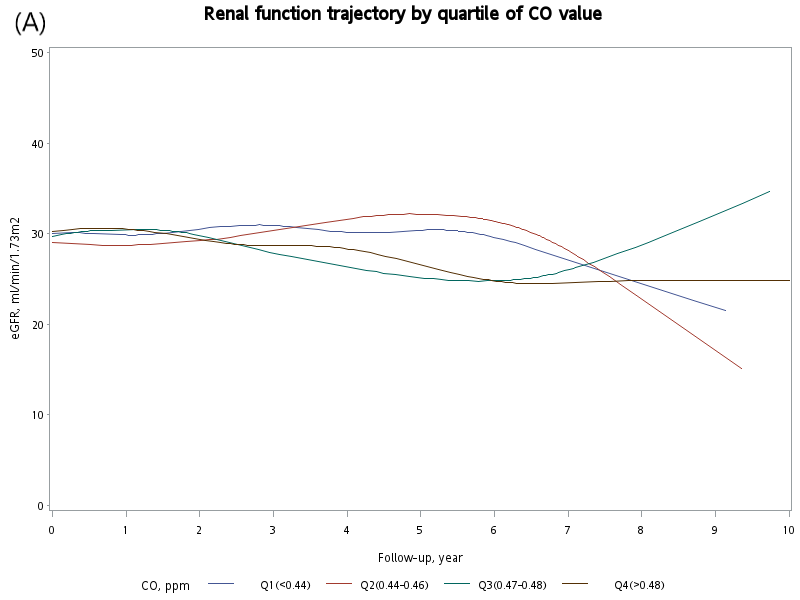


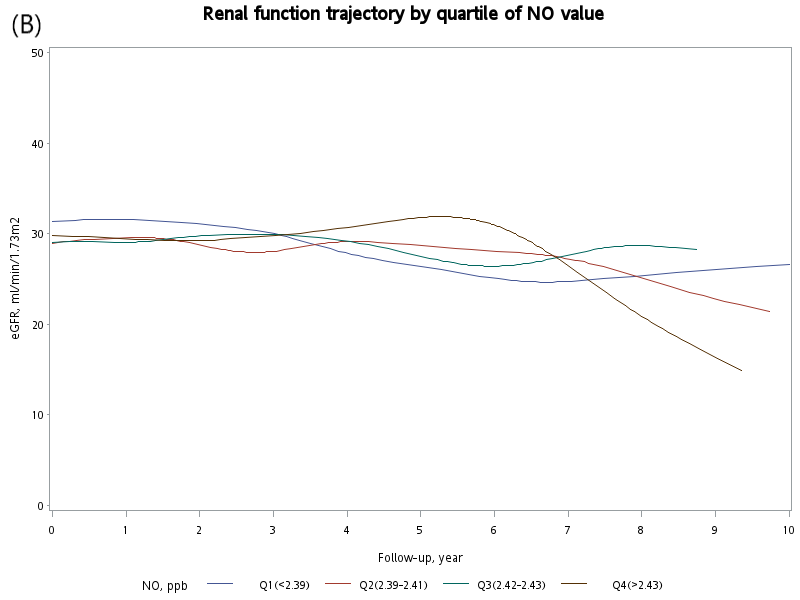


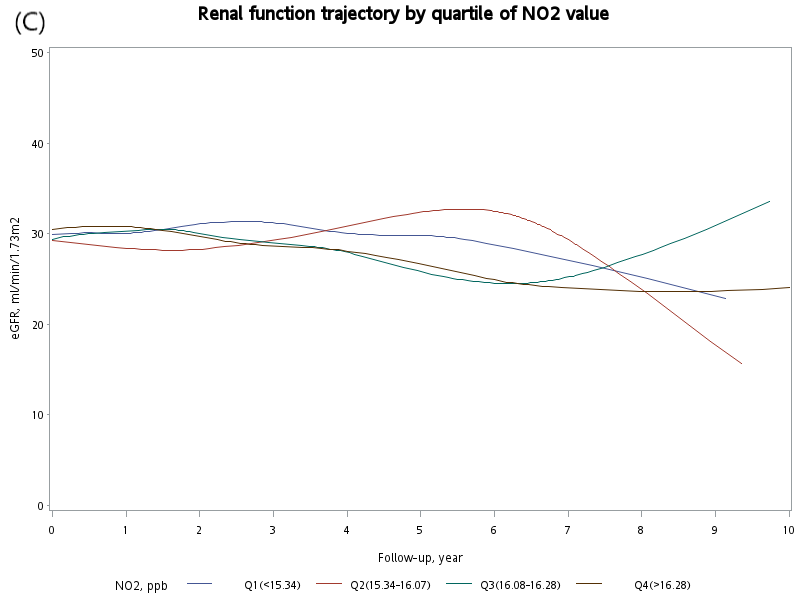


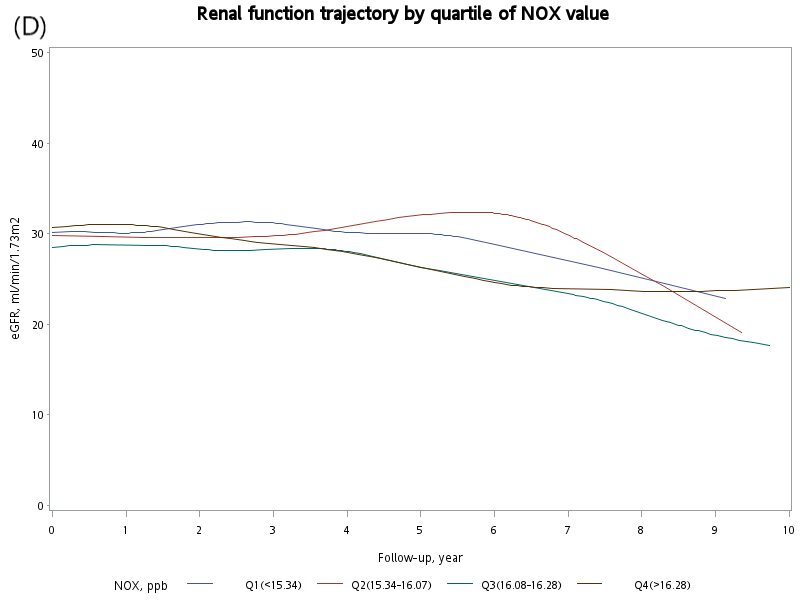


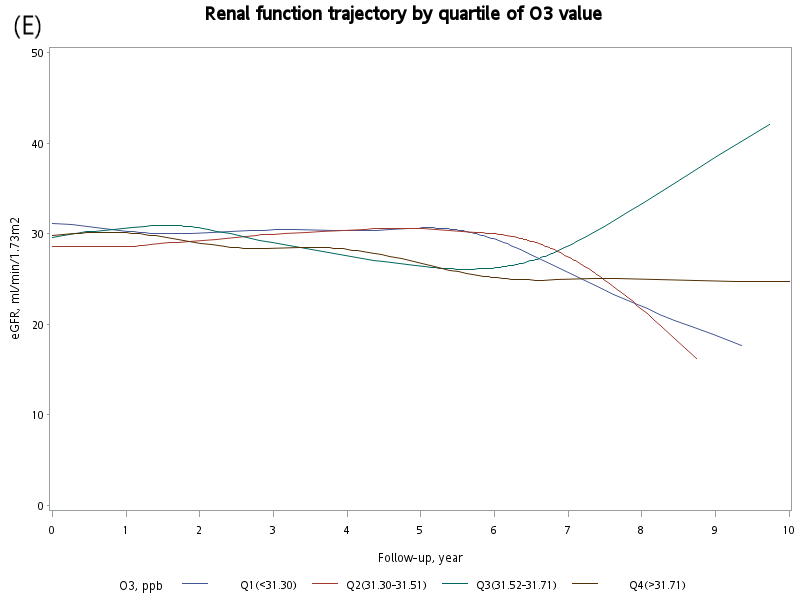


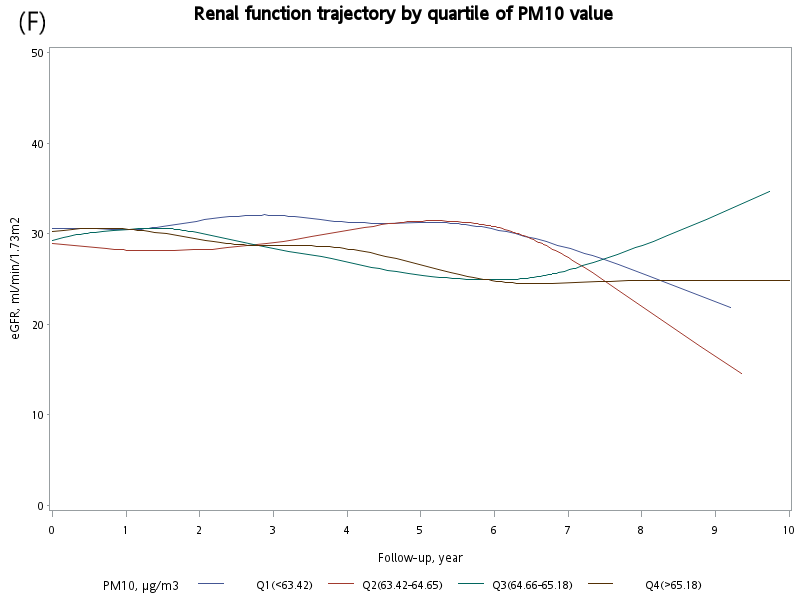


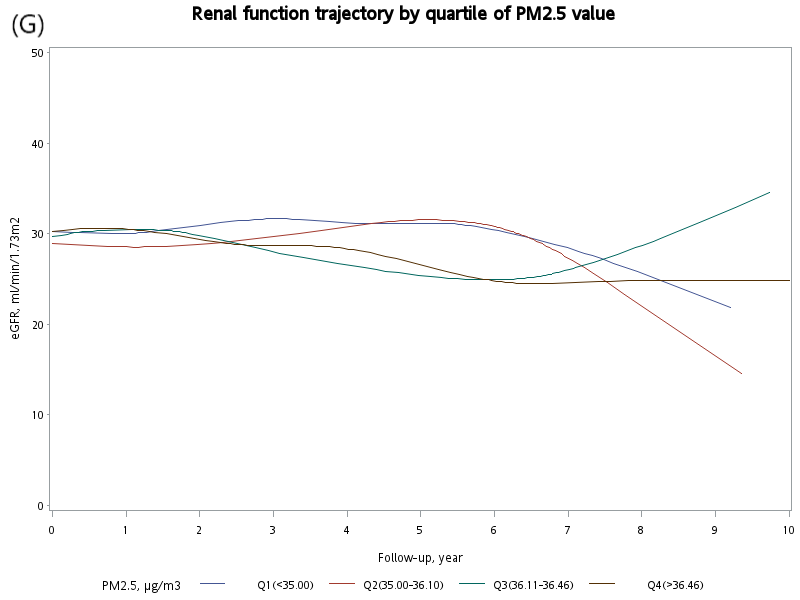


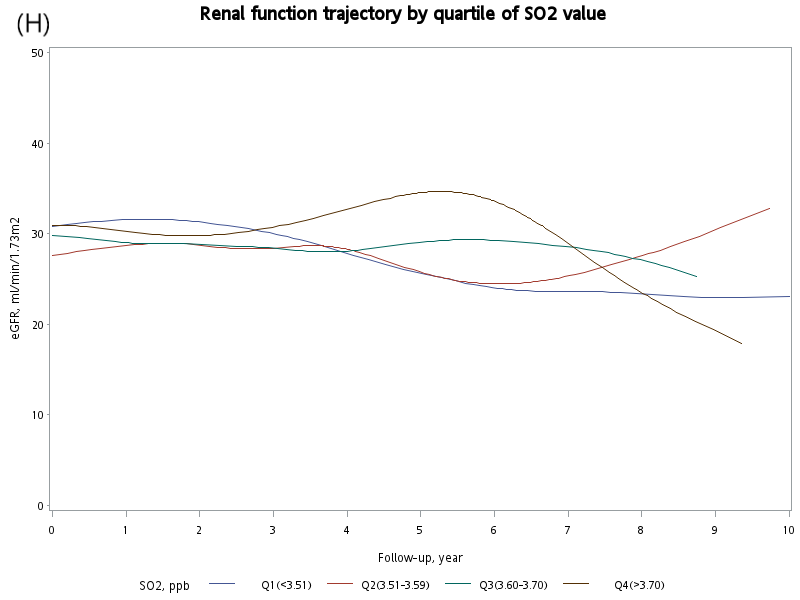

Supplement: Supplementary file 1 — Supplementary Information. [file 41598_2023_36682_MOESM1_ESM.docx]
